# Deciphering the Preference and Predicting the Viability of Circular Permutations in Proteins

**DOI:** 10.1371/journal.pone.0031791

**Published:** 2012-02-16

**Authors:** Wei-Cheng Lo, Tian Dai, Yen-Yi Liu, Li-Fen Wang, Jenn-Kang Hwang, Ping-Chiang Lyu

**Affiliations:** 1 Institute of Bioinformatics and Structural Biology, National Tsing Hua University, Hsinchu, Taiwan, People's Republic of China; 2 Institute of Bioinformatics and Systems Biology, National Chiao Tung University, Hsinchu, Taiwan, People's Republic of China; 3 Department of Biostatistics and Bioinformatics, Emory University, Atlanta, Georgia, United States of America; 4 Department of Life Sciences, National Tsing Hua University, Hsinchu, Taiwan, People's Republic of China; Aston University, United Kingdom

## Abstract

Circular permutation (CP) refers to situations in which the termini of a protein are relocated to other positions in the structure. CP occurs naturally and has been artificially created to study protein function, stability and folding. Recently CP is increasingly applied to engineer enzyme structure and function, and to create bifunctional fusion proteins unachievable by tandem fusion. CP is a complicated and expensive technique. An intrinsic difficulty in its application lies in the fact that not every position in a protein is amenable for creating a viable permutant. To examine the preferences of CP and develop CP viability prediction methods, we carried out comprehensive analyses of the sequence, structural, and dynamical properties of known CP sites using a variety of statistics and simulation methods, such as the bootstrap aggregating, permutation test and molecular dynamics simulations. CP particularly favors Gly, Pro, Asp and Asn. Positions preferred by CP lie within coils, loops, turns, and at residues that are exposed to solvent, weakly hydrogen-bonded, environmentally unpacked, or flexible. Disfavored positions include Cys, bulky hydrophobic residues, and residues located within helices or near the protein's core. These results fostered the development of an effective viable CP site prediction system, which combined four machine learning methods, *e.g.*, artificial neural networks, the support vector machine, a random forest, and a hierarchical feature integration procedure developed in this work. As assessed by using the hydrofolate reductase dataset as the independent evaluation dataset, this prediction system achieved an AUC of 0.9. Large-scale predictions have been performed for nine thousand representative protein structures; several new potential applications of CP were thus identified. Many unreported preferences of CP are revealed in this study. The developed system is the best CP viability prediction method currently available. This work will facilitate the application of CP in research and biotechnology.

## Introduction

Circular permutation of a protein is a structural rearrangement whereby the N- and C-termini of structural homologs are located at different positions. The mechanisms underlying natural CP are not fully understood. Although posttranslational modification may promote CP [1], [2], the majority of CPs result from complex genetic events, such as the proposed duplication/deletion [3], [4], fusion/fission [5], [6] and other models [7], [8], [9]. Since the first observation of CP in plant lectins [1], many naturally occurring CP cases have been documented in well-known protein families (see [10] for a summary). These natural cases led to the conclusion that circular permutants (CPMs) usually retain their native function(s) [4], [11], sometimes with increased functional diversity and/or enzymatic activity [12], [13], [14]. To reveal the effects of CP, many artificial CPMs have been generated. As long as a CP site, *i.e.*, the position for creating the new termini, is not a residue essential for protein folding or function, the artificial CPM generally has native function(s) [4], [15], [16], although its folding pathways and/or the structural stability might be changed [17], [18], [19]. Owning to these discoveries, CP has become a new method beyond traditional mutagenesis for studying proteins [20], [21], [22]. It has also been increasingly applied as a bioengineering technique to modify the stability, solubility and activities of proteins [12], [23], [24], [25]. Particularly, CP allows the covalent linkage of two proteins at positions other than their native termini and has thus made possible the creation of several useful protein switches, molecular biosensors, and novel fusion proteins [26], [27], [28].

Although CP is a powerful technique, its implementation poses a challenge. First, introducing a CP is much more difficult, expensive, and time-consuming than carrying out traditional mutagenesis. Second, not every position in a protein structure can be used to generate a viable (*i.e.*, correctly folded, stable) CPM [25]. Thus, successful application of CP requires selection of an appropriate CP site — a process that is yet ill defined. Currently, researchers who want to engineer protein by CP may have to rely on uneconomic trial-and-error. There has been a general observation that CPs tend to occur at positions with low sequence conservation, high solvent accessibility, and that contribute little to protein folding [29], [30]. A structure-derived residue measure known as “closeness” was applied to predict CP viabilities by Paszkiewicz et al. [30]. Indeed, closeness yielded better results than sequence conservation and solvent accessibility with respect to receiver operating characteristic (ROC) curve analyses. Nevertheless, the area under the ROC curve (AUC) value was only 0.7 [30]. Moreover, because the amount of publicly available data on CP was insufficient at that time, their experimental dataset contained only one protein, dihydrofolate reductase (DHFR), for which the entire polypeptide had been subjected to systematic CP tests [29]. Thus, it was uncertain whether closeness was adequate to predict viable CP sites for other proteins, and to date a practical CP site determination method is still unavailable.

Since the identification of CP and structural comparisons between CPMs are computationally costly [6], [10], [31], [32], CP-related bioinformatics resources were not readily available until 2008 — when the first CP alignment search method, namely CPSARST, was developed [10]. The first semi-manually curated database for CP, namely the CPDB [33], was subsequently established by database searches against the Protein Data Bank (PDB). Later, GANGSTA+, a non-sequential protein structural comparison method, was also applied to large-scale identification of CPs. The present database of GANGSTA+ Internet Services (GIS), a machine-curated database for protein structural homologs, also contains many CPM homologs [34], [35]. The amount of non-redundant CPs recorded in CPDB and GIS nowadays should be sufficient for deciphering the natural preferences of CP in detail. The knowledge of the preferences of CP can help researchers/bioengineers select suitable positions for creating CPMs; it may also help us elucidate the mechanisms of CP and understand how CP sites are selected by nature to enhance protein evolutionary and functional diversity. In order to facilitate fundamental researches and biotech applications of CP, we aim to extensively determine the sequence and structural preferences of CPs and develop an effective viable CP site prediction system in this study.

A major problem in deciphering the preferences of CP and developing CP viability predictors is the lack of information on inviable CP sites (*i.e.*, negative cases). Most wet-lab work only reported viable CPMs. Bioinformatics methods for detecting CP could only identify CPMs that fold into a stable structure. The above-mentioned DHFR dataset contained only 73 negative cases. In our present work, we first established a literature-derived Dataset L consisting of seven proteins with both known viable and inviable CP sites and increased the number of negative cases by 2.4 fold. A 40% sequence identity non-redundant subset of CPDB (nrCPDB-40; containing 1,059 proteins) was also established. Dataset L and nrCPDB-40 were subjected to statistical and ROC curve analyses, seeking to identify sequence, structure and dynamics characters that would discriminate between viable and inviable CP sites. The identified characters and preferences of CP were utilized to develop an elaborate system to predict viable CP sites. Finally, the DHFR dataset and a 40% sequence identity non-redundant subset of GIS (nrGIS-40; 2,814 domains) were used as independent datasets for evaluating the developed prediction system, which achieved an AUC of 0.91 for the DHFR dataset and a large-scale prediction sensitivity of ≥0.72 for either nrCPDB-40 or nrGIS-40. To promote applications of CP, we have applied this system to predict viable CP sites for ∼9,000 representative protein structures and the detailed results are available with the online version of this article.

Our characterization of CP sites was consistent with previous studies and revealed many unreported discriminative properties between viable and inviable CP sites. The CP viability prediction system developed based on these discriminative properties is currently the best among related methods. This work has well achieved its aims to decipher the preference and predict the viability of circular permutations.

## Results and Discussion

### Definition of a CP Site and Determination of a Suitable Representative Segment Size for CP Sites

As illustrated in the Figure S1, a CP site was defined in this study as a position at which two natural structural homologs are related by a CP or a position where an artificial CP was introduced into a protein. If CP was applied to protein *P* with amino acid sequence *A*_1_ *A*_2_ *A*_3_ *A*_4_ *A*_5_ *A*_6_ *A*_7_ to produce protein *P′* with sequence *A*_5_ *A*_6_ *A*_7_ *L*_0_ *A*_1_ *A*_2_ *A*_3_ *A*_4_, then the CP site of *P* is referred to as position 5. Meanwhile, the cleavage point for the CP site will be referred to the peptide bond connecting *A*_4_ and *A*_5_. The *L*_0_ in protein *P′* joining the native N-terminus (*A*_1_) and C-terminus (*A*_7_) is a polypeptide linker, which may or may not be required in an artificial CP. Note that what Figure S1 illustrates and what we describe here is simply a “working definition” of a CP site, rather than the evolutionary mechanism of CP or an actual artificial procedure for creating a circular permutant. Most naturally occurring CP cases are the result of complex genetic events, such as those mentioned in Introduction or summarized in [33]. Similarly, most artificial CPs are created by using delicate genetic-based techniques (see Materials and Methods for references and [24] for a review of current protocols) instead of such a simple polypeptide-based manipulation. We defined a viable CP site as one that led to a foldable and adequately stable CPM, whereas an inviable site led to a CPM that was not foldable or could not be purified. Dataset S1 and the Materials and Methods provide information about how we retrieved known viable and inviable CP sites from literature and established Dataset L (see Dataset S1).

In order to examine the local sequence and conformational propensities of CP sites, in this work CP sites were not studied as individual residues but as polypeptide segments. The nrCPDB-40 dataset with 2,072 viable CP sites was utilized here. Each CP site was temporarily represented by a 20-residue segment (*i.e.*, ±10 residues) surrounding the cleavage point (*p_cut_*). For unbiased analyses, the representative segments were clustered and reduced to a non-redundant subset (nrCPsite_cpdb_-40, consisting of 1,087 CP sites) in which the sequence identity of any two polypeptide was <40% (Materials and Methods). As a preliminary test, bootstrap aggregating analyses were carried out to determine the average occurrence of the 20 amino acids in all proteins in nrCPDB-40 (the comparison/background group) and all *p_cut_*±*k* segments in nrCPsite_cpdb_-40 (*k* = 1, 2, 3 …, 10; the experimental groups). Figure S2 shows that CP has preference for certain amino acid residues. The amino acid occurrence frequencies in the experimental groups increasingly differed from those of the background group as the length of the representative segments was narrowed down to the *p_cut_*. Another preliminary test was done to observe the coverage of occurrence of a variety of sequence and secondary structural element (SSE) patterns for the *p_cut_*±*k* segments. For all patterns a longer segment had a higher occurrence coverage (Table S1); notably, some of the oligo-residue patterns and residue coupling patterns [36] had very low coverage of occurrence or could not even form when *k*<3. Based on these results, we chose *p_cut_*±3 (*i.e.*, 6-residue segment surrounding the cleavage point) to represent a CP site. See Materials and Methods for details.

### Amino Acid Propensities and Physiochemical Preferences of CP

The amino acid preferences of CP have not been characterized previously. This work examined such preferences by utilizing the nrCPDB-40 (background group) and nrCPsite_cpdb_-40 (the CP site group) datasets. Because of the non-normal distribution of the occurrence frequency of many amino acids or sequence patterns in these datasets, Student's t-test was not suitable for the statistical analysis of these data (see Figure S3 for diagrams of the distribution of each amino acid). To analyze whether the average frequency of a sequence pattern around CP sites differed significantly from background, the permutation test — a statistical significance test capable of dealing with non-normally distributed data [37] — was performed to calculate the *p*-value.

**Figure 1:**
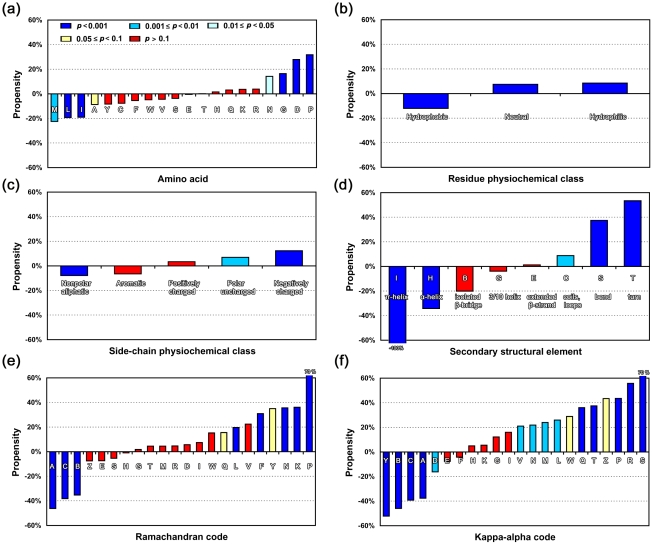
Sequence and Secondary Structural Propensities of Viable CP Sites. In these charts, each bar shows the relative occurrence of a pattern, *e.g.*, an amino acid, a physiochemical type of residue, or an SSE, for the background polypeptides (in dataset nrCPDB-40) and viable CP sites (in dataset nrCPsite_cpdb_-40). The background value was considered as the zero point in each experiment; thus, a positive or a negative value means that the frequency of the pattern at CP sites was higher or lower than its frequency in the background. As shown in chart (a), dark blue- to light blue-colored bars represent smaller *p-*values (<0.05) for the difference between the background and CP site groups. The yellow- and red-colored bars represent *p-*values≥0.05. Patterns examined in this experiment include: (**a**) amino acids, (**b**) residue physiochemical types classified according to [38], (**c**) side-chain physiochemical types classified according to [39], (**d**) SSE determined by DSSP [49], (**e**) Ramachandran code, the backbone conformational alphabet defined by SARST [50], and (**f**) kappa-alpha code, the backbone conformational alphabet defined by 3D-BLAST [51].

As shown in Figure 1, for nrCPsite_cpdb_-40, there was a significant preference for Pro (occurrence ∼32% higher than background) or Gly (∼16% higher) at viable CP sites (*p*<0.001). Hydrophilic residues, especially Asp and Asn (*p*<0.05), were also preferred whereas bulky hydrophobic residues such as Met (*p*<0.01), Leu, and Ile (*p*<0.001) were disfavored (occurrence ∼20% lower than background; Figure 1A). According to [38], we classified amino acids into three groups: hydrophobic, hydrophilic, and neutral. Permutation tests confirmed that hydrophilic and neutral residues had higher occurrence at CP sites whereas hydrophobic residues had lower occurrence (below background) (all *p-*values<0.001; see Figure 1B). We also classified amino acids into five different physiochemical types based on their side chains [39]: nonpolar aliphatic, polar uncharged, aromatic, positively charged, and negatively charged. Viable CP sites preferred negatively charged and polar uncharged residues (12% and 7% above background, respectively; *p-*values<0.01; Figure 1C) and disfavored nonpolar aliphatic residues (8% lower than background; *p*<0.001). Cys and residues with aromatic rings also tended to be disfavored, but the statistical significance for these residue types was low (*p*>0.1) for nrCPsite_cpdb_-40.

GIS [35] includes domain-based “partial” CPs [10], an aspect that differs from CPDB, which strictly considers the entire polypeptide chain as the unit of CP [33]. As a result, nrCPsite_gis_-40 — a non-redundant CP site dataset of GIS (see Materials and Methods) — contained 2.5 times as many CP sites as nrCPsite_cpdb_-40. A repetition of the above experiments with nrCPsite_gis_-40 yielded similar tendencies as for nrCPsite_cpdb_-40 (refer to Figure S4). Based on nrCPsite_gis_-40, we confirmed that Cys and aromatic residues were relatively disfavored for CP (11% and 10% lower occurrence than background, respectively; *p*<0.05; Figure S4A and S4C). Further, analyses with both datasets confirmed that CP had little or no preference for positively charged residues (Figure 1C and S4C). Permutation test-based compositional analyses of di-residue, oligo-residue, and coupling-residue [36] patterns were also performed. See Figure S5 for details.

CP prefers Pro, Gly, and hydrophilic residues and disfavors bulky hydrophobic residues. These results can be explained by earlier findings. The fact that viable CPs prefer positions with relatively low sequence conservation and high solvent accessibility [29], [30] implies they favor loop conformations, which are usually less conserved in protein families [40] and are generally exposed to the solvent [41]. Indeed, Pro and Gly are frequently found in loops [42]. Besides, hydrophilic residues are more solvent exposed than hydrophobic residues, among which the bulky ones are more frequently buried in proteins (see the second table of [43]). Moreover, Cys residues, particularly those involved in disulfide bridges, are important for protein folding/stability [44], [45]. Occurrence of a CP at a disulfide-bridged Cys prevents formation of the disulfide bond, and thus it would be expected that viable CPs would seldom occur at disulfide bond-forming Cys residues.

The negatively charged Asp and hydrophilic Asn were much more favored at CP sites than residues with similar physiochemical properties, *i.e.*, Glu and Gln. They were also preferred over the positively charged (and hydrophilic) residues Arg and Lys. These seemingly obscure results might have biological significance because they were independently observed in CPDB and GIS. Although at present these differences in propensity cannot be easily rationalized with our limited knowledge of sequence-structure relationships in proteins, the observed CP preference for Asp and Asn over Glu, Gln, Arg, and Lys is just the reverse of the preference of these residues in helices [46], [47], [48]. Taken together, our sequence-based statistics suggest that natural CPs prefer positions in loops whereas positions in helices are disfavored.

### Secondary Structure Preferences for CP

The putative secondary structure preferences for CP deduced above were supported by secondary structural propensity analyses. In this work, polypeptide secondary structure was determined by the program DSSP, which categorized secondary structure into eight types [49]. Figure 1D shows that residues within turns, bends or loops/coils were greatly preferred over those within α- or π-helices or β-bridges. Extended β-strands and 3/10 helices showed no preference for CP over background. Several structural alphabets have been developed to describe local backbone conformation in polypeptides, among which the Ramachandran codes developed by Lo et al. [50] and the kappa-alpha codes of Yang et al. [51] correlate backbone conformations with two-dimensional Ramachandran and kappa-alpha plots and are thus easy to visualize. Using the Ramachandran codes to describe protein backbone conformations (see Figure 1E), we found that CP particularly favored the codes that corresponded to the Ramachandran plot regions with high populations of isolated β-strands, random coils, turns, and Pro residues (refer to Figure S6 and [52]), the last of which was consistent with our sequence-based results. CP sites occurred infrequently at residues located in Ramachandran code regions A–C (35–46% lower occurrence than background), which are exclusively occupied by α-helices [50], [52]. The Ramachandran code only describes a 3-residue backbone conformation. To convey longer backbone conformations, we utilized the kappa-alpha code, which encodes 5-residue backbone conformations [51]. Similarly, CPs occurred rarely at residues located in the kappa-alpha code regions of helical conformations (Figure 1F; see also Figure S6 and [51]). We also analyzed several di-residue, oligo-residue, and coupling-residue secondary structural patterns; CP occurred frequently at the terminal residues of regular SSEs, *i.e.*, helices and strands. The occurrence of CP at transitional regions between a bend and a β-strand, between a turn and a β-strand, between an α-helix and a coil, etc., were much higher than the background occurrence of these di-residue SSE patterns (summarized in Figure S5).

CP highly favors coils, loops, and turns, and highly disfavors helices and β-bridges. Again, the conformations preferred by CP are evolutionarily less conserved and/or are generally located at positions with higher solvent accessibility than the non-preferred conformations. Inferring from these preferences, viable CP sites are more likely to be: (1) located closer to the protein surface, (2) less intra-molecularly hydrogen-bonded, because coils and loops have less-well-defined hydrogen-bonding patterns than regular SSEs, and (3) more flexible than inviable CP sites.

### Definition of the Propensity Score and the Distribution of Propensity Scores Based on Sequence and Secondary Structural Information

So far our sequence and secondary structure statistical results agreed well with each other and were biologically relevant. Before further investigating the preferences of CP, we tested whether the current information was sufficient to distinguish viable from inviable CPs. A new propensity scoring system was defined as follows,
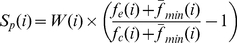
(1.1)

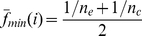
(1.2)

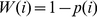
(1.3)where *S_p_* is the propensity score, *i* is the sequence/SSE pattern under analysis, *f_e_*(*i*) and *f_c_*(*i*) are the frequencies of occurrence of pattern *i* in the experimental and comparison groups, respectively, *n_e_* and *n_c_* represent the amounts of pattern *i* in the two groups, and *p*(*i*) is the *p-*value of *i* obtained by the permutation test. The 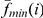 is defined as the average of the minimum frequency of occurrence of *i* in both groups. The fundamental purpose of using 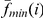 in Formula 1.1 is to prevent dividing by 0. *W*(*i*) is a weighting function, which reduces the value of a score if the statistical significance of the data is low. A positive or negative *S_p_* score indicates that *i* has a higher or lower occurrence, respectively, in the experimental group than in the comparison group. A zero *S_p_* means that either *i* has an equivalent frequency in both groups or the results have extremely low statistical significance.

**Figure 2:**
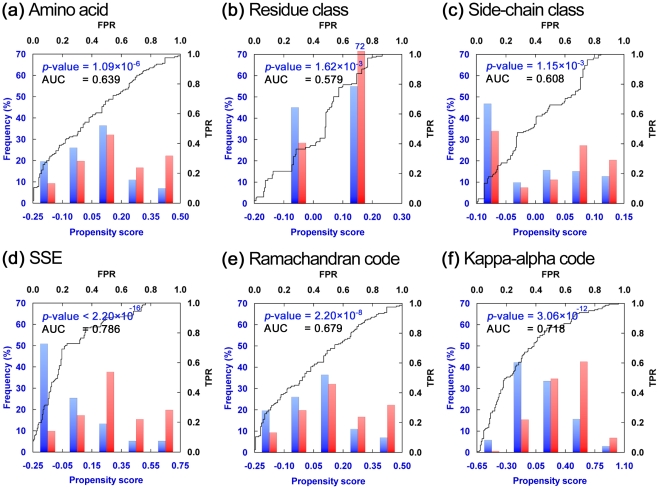
Distributions and ROC Curves of Propensity Scores. Here, a propensity score was calculated as the relative propensity of a pattern between the background and viable CP sites weighted by 1 – *p-*value (see Formula 1). A high relative propensity and a small *p-*value resulted in a high score. A zero score means that there was no obvious difference between the frequencies of the pattern in the background and viable CP sites, or the difference was statistically insignificant. These plots show distributions of several propensity scores for the viable (red bars) and inviable (blue bars) CP sites of Dataset L and their ROC curves. Plots (**a**)–(**c**) and (**d**)–(**f**) respectively exhibit the results of sequence-based and secondary structure-based propensity scores. The distributions of the sequence-based propensity scores are not very different between the viable and inviable CP sites, and their AUCs are only ∼0.6. The distributions of secondary structure-based propensity scores were rather different between viable and inviable CP sites, and thus the AUCs were higher than those of sequence-based scores. The lower *x* axis in each plot indicates the propensity score. The left *y* axis indicates the frequency, *i.e.*, the proportion of residues falling into each score group. The upper *x* axis and right *y* axis represent the false positive rate and true positive rate, respectively, for the ROC curve.

The feature of this scoring system is that it takes statistical significance into consideration to ensure the reliability of the scores. After applying this system to nrCPDB-40, we obtained the propensity scores of many sequence/SSE patterns. To evaluate these scores, t-tests and ROC curve analyses were performed on Dataset L. The distributions of most propensity scores differed significantly between viable and inviable CP sites (Figure 2). For instance, the *p-*values of the amino acids and SSE propensity scores were 1.1
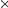
10^−6^ and <2.2
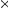
10^−16^, respectively. The sequence-based propensity scores achieved a binary classification power (AUC = ∼0.60) similar to the solvent accessibility measure used by Paszkiewicz et al. (AUC = 0.58) [30]. Notably, the secondary structure-based propensity scores registered an average AUC of 0.73, comparable to the classification performance of closeness (AUC = 0.7) [30]. The source dataset (nrCPDB-40) of the statistics used to calculate propensity scores and the evaluation dataset (Dataset L) shared <40% sequence identities. In addition to demonstrating that the proposed propensity scoring system is feasible to develop a CP site prediction procedure, these results suggested that our sequence and secondary structural statistical results properly reflected the natural preferences of CP.

### Solvent Accessibility and Depth of CP Sites

**Figure 3:**
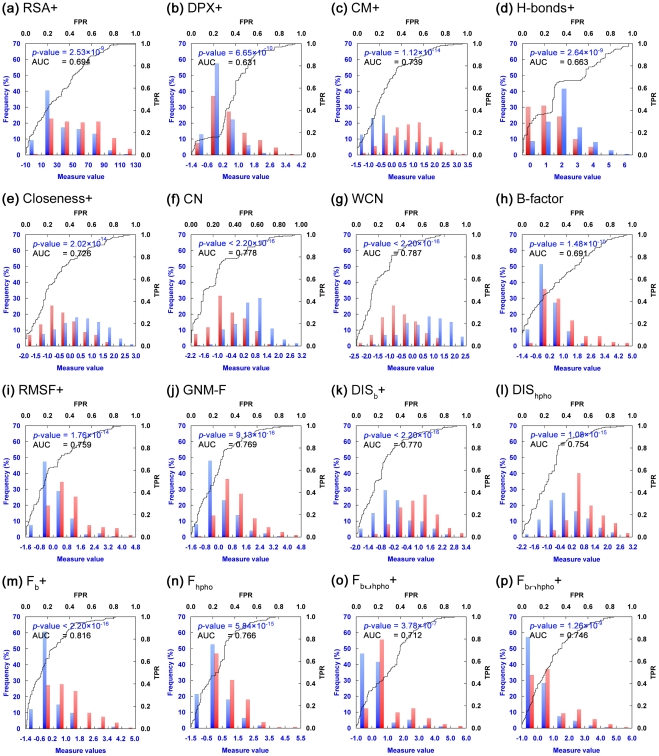
Distribution and ROC Curves of Various Tertiary Structure-derived Residue Measures. In general, the differences in the distributions of tertiary structure-derived residue measures in viable (red bars) and inviable (blue bars) CP sites of Dataset L were larger and statistically more significant than those of the sequence and secondary structural propensity scores. Their AUC values were also larger in most cases. See Figure 2 for descriptions of the four axes. The abbreviations shown on top of each plot stand for: (**a**) relative solvent accessibility, (**b**) residue depth, (**c**) centroid distance measure, (**d**) number of hydrogen bonds, (**e**) closeness, (**f**) contact number, (**g**) weighted contact number, (**h**) atomic mean-square displacement, (**i**) root-mean-square fluctuation of the Cα atom, (**j**) Gaussian network model-derived mean-square fluctuation, (**k**) average distance to the residues located in the buried core, (**l**) average distance to hydrophobic residues, (**m**) “farness” (see the main text for definition) from the buried core, (**n**) farness from hydrophobic residues, (**o**) farness from the union set of residues in the buried core and hydrophobic residues, and (**p**) farness from the hydrophobic residues located in the buried core. A plus (+) after an abbreviation for certain measures indicates that hydrogen atoms were restored/added before those measures were calculated. If the definition or algorithm of a measure did not consider hydrogen atoms, or if it made no difference to the results whether hydrogen atoms were present, that measure was computed without adding hydrogen atoms.

The relative side-chain area defined as the molecular surface area inaccessible to solvent molecules is a measure complementary to solvent accessible surface area [30]. This measure has been applied to predict viable CP sites (AUC for DHFR = 0.58 [30]). Here we utilized the standard solvent accessibility measure RSA (relative solvent accessibility) [53], [54], [55], to study CP. As shown in Figure 3A and Table 1, CP significantly prefers residues with high solvent accessibility (*p*-value = 2.5
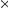
10^−9^), and RSA is feasible to distinguish viable from inviable CP sites (AUC = 0.69).

**Table 1:** Binary Classification Performance of Several Propensity Scores and Tertiary Structure-derived Residue Measures.

Score/Measure^a^	Viable CP sites^a^	Inviable CP sites^a^	*p-*value	AUC	Decision threshold^b^	Sensitivity	Specificity	MCC
R_aa	0.062±0.155	−0.016±0.129	1.09 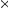 10^−6^	0.639	0.000	0.574	0.618	0.193
R_aat3	0.023±0.091	−0.011±0.100	1.62 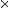 10^−3^	0.579	0.075	0.716	0.462	0.184
R_aat5	0.015±0.081	−0.013±0.076	1.15 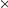 10^−3^	0.608	0.023	0.586	0.590	0.176
R_sse	0.162±0.257	−0.114±0.263	<2.20 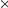 10^−16^	0.786	0.087	0.728	0.769	0.498
R_rm	0.126±0.270	−0.059±0.319	2.20 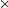 10^−8^	0.679	0.062	0.623	0.676	0.300
R_ka	0.165±0.292	−0.077±0.319	3.06 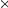 10^−12^	0.718	0.037	0.698	0.653	0.351
RSA+	46.602±29.258	27.698±26.846	2.53 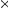 10^−9^	0.694	26.000	0.710	0.572	0.284
DPX+	−0.487±0.628	0.116±1.053	6.65 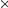 10^−10^	0.631	−0.766	0.667	0.595	0.262
CM+	0.586±0.914	−0.238±0.945	1.12 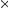 10^−14^	0.739	−0.112	0.778	0.642	0.422
H-bonds+	1.284±1.141	2.064±1.184	2.64 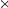 10^−9^	0.663	−2.000	0.648	0.688	0.336
Closeness+	−0.551±0.832	0.247±0.983	2.02 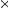 10^−14^	0.726	−0.155	0.716	0.665	0.381
CN	−0.643±0.901	0.359±0.938	<2.20 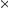 10^−16^	0.778	−0.050	0.759	0.723	0.482
WCN	−0.709±0.788	0.337±0.998	<2.20 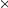 10^−16^	0.787	−0.060	0.815	0.676	0.495
B-factor	0.418±1.127	−0.267±0.674	1.48 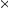 10^−10^	0.691	−0.017	0.568	0.699	0.270
RMSF+	0.546±1.115	−0.324±0.817	1.76 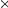 10^−14^	0.759	−0.135	0.698	0.705	0.403
GNM-F	0.594±1.026	−0.309±0.912	9.13 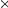 10^−16^	0.769	−0.127	0.772	0.705	0.477
DIS_b_+	0.618±0.864	−0.317±0.948	<2.20 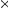 10^−16^	0.770	0.020	0.741	0.717	0.457
DIS_hpho_	0.499±0.808	−0.302±0.926	1.08 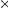 10^−15^	0.754	0.000	0.821	0.671	0.496
F_b_+	0.649±1.090	−0.378±0.774	<2.20 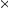 10^−16^	0.816	−0.247	0.765	0.711	0.477
F_hpho_	0.443±0.871	−0.315±0.814	5.84 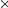 10^−15^	0.766	−0.077	0.778	0.682	0.461
F_b_∪_hpho_+	0.505±1.284	0.129±0.889	3.78 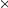 10^−7^	0.712	−0.368	0.747	0.595	0.346
F_b_∩_hpho_+	0.520±1.222	−0.218±0.884	1.26 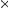 10^−9^	0.746	−0.436	0.759	0.636	0.397

a The values of these measures are all presented here with the format: mean ± standard deviation. A plus (+) after an abbreviation for certain measures indicates that hydrogen atoms were restored/added before those measures were calculated. See Figures 2 and 3 for the meaning of abbreviations used for these measures.

b For convenience, the optimal decision threshold of a score was determined as the score value corresponding to the point nearest to point (0,1) on the ROC curve [98]. The sensitivity, specificity and MCC were obtained at the listed decision thresholds.

The relatively higher RSA values of viable CP sites indicate that they are located closer to the surface of protein. We used two measures, residue depth and centroid distance measure (CM), to verify this inference. The residue depth was the depth of a residue measured from the solvent-accessible surface [56]. In this study, the location of a residue was represented by its alpha carbon atom (Cα), unless otherwise specified. The CM value of a residue was defined as the distance of its Cα from the protein's center of mass [57], [58].

Indeed, the distributions of residue depth and CM values confirmed that viable CP sites were, on average, closer to the solvent-accessible surface and farther away from the center of mass (Figure 3B and 3C). The performance of using residue depth to distinguish viable from inviable CP sites was lower than that of RSA, whereas CM was better than RSA in this regard; the AUCs for residue depth and CM were 0.63 and 0.74, respectively. Because residue depth and CM measure different attributes of a residue, the observed difference of their binary classification performance implied that the actual cause of the preference of CP for positions close to the solvent-accessible surface might be a lower viability of a CP occurring at a position close to the central buried region of the protein. See Subsection “Farness of CP sites from the buried core of the protein” for further discussion of this matter.

### Number of Hydrogen Bonds and Local Packing Density of CP Sites

Hydrophilic residues (preferred by CP) have higher chances to form intra-molecular hydrogen bonds compared with hydrophobic residues. However, loops and coils (also preferred by CP) are less hydrogen-bonded than regular SSEs. We supposed that the secondary structural property of a residue may play a more dominant role than its hydrophilicity for determining its viability for CP, and thus hypothesized that viable CP sites might be less intra-molecularly hydrogen-bonded. Indeed, 61% of viable CP-site residues formed one or no intra-molecular hydrogen bond whereas 71% of the inviable CP sites had two or more (Figure 3D).

We next hypothesized that, in addition to hydrogen bonds, CP viability could be affected by other intra-molecular interactions such as disulfide bonds, electrostatic forces, and hydrophobic effect. We also presumed that a residue subjected to relatively more attractive intra-molecular interactions would have a more packed (crowded) neighborhood. Generalizing from the fact that viable CP sites make fewer hydrogen bonds, they may also prefer a less packed local environment and/or have fewer (attractive) interactions with its neighboring residues. Several structurally derived residue properties have been applied to describe the local packing density of a residue and/or the density of possible interactions associated with it, including closeness, contact number (CN), and weighted contact number (WCN). If we consider a protein structure as a graph consisting of nodes and edges in which a node represents a residue and an edge represents any possible interactions between two residues that can happen within a specific distance cutoff, then closeness measures the proportion of nodes that can be traversed through an edge from a specific node [30], [59]. Hence, a residue with a higher closeness value has more neighboring residues with which it can interact directly or indirectly [59]. The general definition of CN is the number of atoms surrounding a residue within a sphere of specified radius from its Cα. Previous studies applied various radii (from 6 to 20 Å) in different circumstances [60], [61], [62], [63]. Regardless, a larger CN implies that a residue has more surrounding atoms. For WCN, a weight inversely proportional to the square of distance was given to a surrounding atom *j* of the residue of interest *i* as *j* was counted into the CN of *i* [64]. Residues having many close neighboring residues would thus have a large WCN.

Viable CP sites significantly have smaller closeness, CN and WCN values when compared with inviable sites (*p*-values<2.0
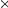
10^−14^). The AUC of closeness was 0.73 as assessed with Dataset L (Figure 3E), consistent with [30]. For CN, the best discriminating capacity was achieved with a radius of 6.4 Å (AUC = 0.78; Figure 3F). WCN could easily distinguish between viable and inviable CP sites (AUC = 0.79; Figure 3G). These results suggested that CP favors positions with a less packed environment. Further, since CN and WCN have been shown to be well correlated to the B-factors [63], [64], our results indicated that CP appears to prefer flexible residues.

### Flexibility of CP Sites

Our data thus far indicated that WCN, CN, and CM were the best structural descriptors for identifying viable CP sites. Notably, all three of them had previously been correlated with residue flexibility; residues with high B-factor values, *i.e.*, highly flexible, usually had small WCN and CN values (unpacked environment) [63], [64] and high CM values (close to the protein surface) [57], [65]. This is reasonable because a residue located in a highly packed region was unlikely to move or rotate freely without affecting its neighboring residues. Combined with our findings that CP favors residues with high RSA values and loop/coil conformations, it seemed reasonable to assume that residues amenable to CP would be those having relatively high flexibility because RSA has also been correlated with residue flexibility [66], and loops/coils are generally flexible.

All protein structures in Dataset L were determined by x-ray crystallography, and hence their B-factors are available. As revealed by Figure 3H, the distribution of B-factors differed significantly (*p* = 1.5×10^−10^) between viable and inviable CP sites, the former having higher values. ROC curve analysis also confirmed that B-factors could reasonably discriminate viable from inviable CP sites (AUC = 0.69). A practical problem of using B-factors is that they are only available for x-ray crystal structures. To make residue flexibility widely accessible, we performed molecular dynamics (MD) simulations on each protein in Dataset L for 100 ps and calculated the root mean square fluctuation (RMSF) for each residue. Residues with high RMSF are more flexible [67]. The distribution of RMSF showed that viable CP site residues had higher RMSF than inviable ones (*p* = 1.8×10^−14^; Figure 3I). The AUC obtained by RMSF was 0.76, clearly better than that by B-factor. However, computing RMSF via MD simulations has an extremely high computational cost, thus limiting the practical application of this approach. A more CPU-efficient Gaussian Network Model (GNM), which is based on a coarse-grained elastic network model, has often been used to probe structure dynamics [68], [69], [70]. As revealed by Figure 3J, the GNM-derived mean-square fluctuation (GNM-F) had a more significant distributional difference (*p* = 9.1×10^−16^) and better discriminating power than RMSF (AUC = 0.77); this AUC was comparable to that of CN or WCN.

With these results coming from crystallographic data, MD simulations, or the theoretical model, we have shown that CP prefers positions with higher flexibility. The high statistical significance and binary classification quality achieved by the residue flexibility measures utilized here and the packing density measures discussed above suggested that flexibility and packing play important roles in determining CP viability. The fact that these measures performed similarly is consistent with the results of previous studies that flexibility can be inferred from local packing density [63], [64].

### Definition of the Farness, a New Residue Measure

Experimental results related to the structure-derived properties examined above emphasized that CP favors residues in a less packed environment and positions farther from a protein's center of mass. For a globular protein, the center of mass is most often located at the very interior (*i.e.*, the core) of the structure, which generally is quite hydrophobic and solvent inaccessible. These attributes of interior residues correlate with the non-preferences of CP as determined by our study. For a highly globular protein, CM alone should be sufficient to distinguish viable from inviable CPs. Nonetheless, most proteins are not totally globular, and in some cases the center of mass may even be outside the protein structure (as a fact in physics, the center of mass of an object may not correspond to any position within the object). We speculated that it is actually the residues close to the hydrophobic/buried core that constituted the sites disfavored for CP. Thus, compared with CM, a measure that could more precisely describe the distance from a given residue to the hydrophobic/buried core might be more feasible to determine the CP viability of the residue.

The hydrophobic/buried core is not a single point but a region. To measure how distant a residue is from the core, we calculated the distance between the residue and the average position of Cα of the residues in the core (see Table 1 and Figure 3K for evaluations). However, we presumed that a core residue *j* located closer to the residue of interest *i* would exert a larger influence. In this case, the harmonic mean of the distances would apply, that is,
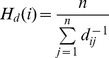
(2)where *d_ij_* is the distance between *i* and *j*; *n* denotes the number of residues contained in the hydrophobic/buried core. If we only considered the relative values among residues, the constant *n* could be omitted. Finally, we added some weight to each distance and generalized the idea into a new “farness” measure (*F*), which described the distance between any residue *i* and a specific group of residues *G*:
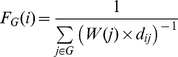
(3)where *W*(*j*) denotes the weight function for each residue *j* belonging to *G*. By referring to the design of WCN, we inferred that a suitable weight function for this farness measure could be 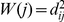. In the general case, *G* can be any target group with a specific property. In this work, *G* represented the buried and/or hydrophobic core (see below for the determination of *W* and working definitions of *G*).

### Farness of CP Sites from the Buried Core of the Protein

Before the “farness” measure could be applied, we needed to identify the group of residues (*G*) constituting the buried and/or hydrophobic core. We supposed that the region defined by all hydrophobic residues in the protein would include the hydrophobic core, and that the core of a protein structure should be composed of residues essentially solvent inaccessible. These two suppositions led to four working definitions of *G*, *e.g.*, (1) set *B*: buried residues (each with RSA<10%); (2) set *H*: hydrophobic residues [38], (3) *B* 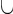 *H*, and (4) *B* 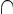 *H*. Among these sets, farness from *B* achieved the best binary classification quality (Table 1; Figure 3M–P). Set *B* actually represented the buried core of the protein, but it was not yet clear whether it was the buried core or hydrophobic core that was less preferred by CP because the residues comprising the hydrophobic core are not as definable as those in the buried core and we presumed that none of the other three sets could perfectly define them.

We assigned F_b_ to represent the “farness” measured from the buried core. After many tests, the working weight function for Formula 3 was actually determined as 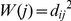 for F_b_. The distribution of F_b_ between viable (higher F_b_) and inviable CP (lower F_b_) sites was very significant (*p*<2.2×10^−16^; Figure 3M). Impressively, the binary classification performance of F_b_ (AUC = 0.82) was clearly better than any other measure we assessed.

The high discriminating power of F_b_ supported our latest hypothesis that residues in the buried core are far less likely to undergo CP. This phenomenon may be explained based on energetics. It seems energetically disfavored to introduce a CP into the core of a protein structure because by doing so the new terminal residues, which were originally buried in a very hydrophobic environment, would probably be exposed to the hydrophilic solvent, not to mention that the dramatic conformational difference between the native structure and the partially “inside-out” permuted structure might require completely different folding pathways. Because F_b_ is a simple measure of distance and cannot be directly applied to describe energetics, we thus proposed that if some thermodynamic measure(s), such as ΔΔG, related to the expected conformational difference between the native and a permuted structure could be properly determined or simulated, the viability of CPs might be more precisely predicted. We also expected that, a cleavage site resulting in a higher ΔΔG would be a less preferred CP site. Clearly, this hypothesis must be tested.

### Predicting CP Viability by Integrating Primary and Secondary Structural Propensity Scores with Tertiary Structural Property Measures

#### Usages of Various Datasets

To prevent dataset overfitting and biased prediction, during the development of prediction procedures discussed in the following text, several datasets were carefully used. Dataset L was divided into two subsets, Dataset T and the DHFR dataset (see Dataset S1), which shared <9% sequence identities (calculated by FASTA [71]). Dataset T and nrCPDB-40 were employed to train/test a predictor. The DHFR dataset and nrGIS-40 were utilized as independent datasets for evaluating the generated predictors. Any two of these training/testing and independent datasets shared <40% sequence identities.

#### Preliminary Feature Adjustments and Selections

There were 48 primary structural propensities, 19 secondary structural propensities, and 36 tertiary structural properties examined in this study, inclusive of a negative control tester for the prediction methods (*e.g.*, random values). These features had various ranges of values. To ensure that viable CP sites had uniformly higher scores in our prediction system, as indicated in Table S2 any feature *M* with lower values among the viable CP sites compared with the inviable sites was either inversed (1/*M*) or multiplied by minus one (−*M*). Next, all features were standardized by the following standard score function,
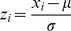
(4)where *i* is the residue of interest, *z* denotes the standard score, *x* is the raw score, and *μ* and *σ* are the mean and standard deviation of scores for all residues in the protein.

From these 103 features, 46 were chosen for the feature set for developing prediction procedures. The choices were made based on the statistical significance, binary classification qualities, redundancy, and ease of implementation of all features (see Table S2 for details). The dataset independence of each selected feature was ensured by establishing a single-feature predictor that was trained and tested with very different datasets — Dataset T and nrCPDB-40. Detailed procedures for assessing the dataset independence of various features are described in Materials and Methods. The prediction performances of single-feature predictors are summarized in Table S3.

#### Hierarchical Integration (HI) of Features

**Figure 4:**
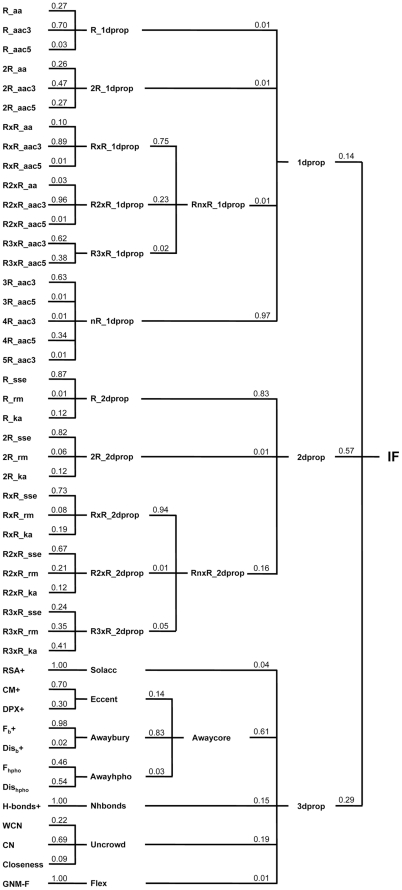
Classification Tree of the 46 Selected Features. These features were selected based on their discriminatory performance for viable and inviable CPs in Dataset T. Redundant features (correlation coefficient >0.7) were screened out. The classification was done manually according to the similarities of biological meaning of these features. The purpose of this classification was to perform the hierarchical feature integration procedure developed in this work. The number following each feature abbreviation was the weight of that feature used in the hierarchical integration procedure. These weights were determined with the training Dataset T by exhaustive performance screening (**Materials and Methods**). Table S2 lists the complete meanings of the features abbreviated here.

Forty-six selected features were quite many. An HI procedure was thus designed to incorporate the binary classification power of various features into a single score (see Materials and Methods and Figure 4). Features were hierarchically classified into a tree-like structure. The feature scores of the branches were averaged with weights into the integrated feature (IF) score of their common node. Finally, a root IF score was produced. This HI procedure was easy to implement, and the root IF could effectively separate the viable and inviable CP site groups of Dataset T by ∼1.2 standard deviations (*p*<2.2×10^−16^). The 10-fold averaged AUC and MCC of this final IF for Dataset T were 0.83 and 0.49. Applying the HI model trained with Dataset T to predict viable CP sites in proteins of nrCPDB-40, the sensitivity was 0.73 (Table 2).

**Table 2:** CP Viability Prediction Performance of Various Procedures.

Dataset	Performance measure	Closeness+^a^	Farness: F_b_+^a^	HI	ANN	RF	SVM	Combined^b^
Dataset T^c^	AUC	0.746	0.768	0.828	0.885	0.844	0.819	0.905
	Sensitivity	0.577	0.761	0.771	0.852	0.775	0.647	0.857
	Specificity	0.677	0.569	0.723	0.846	0.714	0.762	0.790
	False positive rate	0.323	0.431	0.277	0.154	0.286	0.238	0.210
	MCC	0.264	0.329	0.490	0.690	0.483	0.407	0.632
DHFR	AUC	0.814	0.873	0.843	0.833	0.840	0.822	0.906
	Sensitivity	0.465	0.849	0.616	0.593	0.733	0.605	0.709
	Specificity	0.918	0.740	0.918	0.863	0.808	0.918	0.918
	False positive rate	0.082	0.260	0.082	0.137	0.192	0.082	0.069
	MCC	0.421	0.594	0.551	0.467	0.539	0.541	0.633
nrCPDB-40	Sensitivity	0.622	0.616	0.733	0.735	0.733	0.778	0.746
nrGIS-40	Sensitivity	0.614	0.590	0.700	0.682	0.698	0.715	0.715

a Random forest was applied in this experiment to the assess the prediction power of closeness and farness.

b A combination of the four machine learning methods (HI, ANN, RF and SVM) by averaging their probability scores into a single score. See the main text for details.

c These results were obtained with 10-fold cross-validation.

To this point, we had used Dataset T and nrCPDB-40 in the feature selection step and utilized them again to train and evaluate the prediction method. To avoid biased evaluations, independent datasets were used to ultimately assess the performance of the HI procedure. The AUC and MCC for the DHFR dataset were respectively 0.84 and 0.55 and the sensitivity for nrGIS-40 was 0.70. Hence, the HI procedure was appropriate for predicting the viability of CP sites.

### Prediction of CP Viability Using Machine Learning Techniques

In additional to the HI procedure, three well-developed machine learning techniques, ANN (artificial neural networks), SVM (support vector machine), and RF (random forest), were also applied. In every method, the output for each residue was designed to be a probability score for its being a viable CP site (Materials and Methods). Trained with the same 46 features and training datasets utilized in the previous subsection, each of these techniques performed well. As shown in Table 2, all of them achieved high AUC (>0.82) and MCC (>0.46) values on the independent dataset DHFR. The large-scale prediction performances of these methods on the independent dataset nrGIS-40 were also acceptable (all sensitivity values were >0.68).

The four methods had different properties. We supposed that combining the prediction power of them would improve the performance and further decrease dataset overfitting. Thus, for each residue subjected to our final prediction system, the probability scores computed by the four methods were averaged into a single score. If the input residue had a final probability score ≥0.5, it was predicted as a viable CP site.

Before this work, the best CP viability prediction methods were developed based on the closeness measure, which registered an AUC of 0.7 on the DHFR dataset [30] and a sensitivity of 0.67 on CPDB [33]. The performance of the prediction system developed here was greatly improved. As shown in Table 2, the AUC obtained by our combined machine learning system on the DHFR dataset was 0.91, and the sensitivity on nrCPDB-40 was 0.75. In addition, the sensitivity value of this system on the independent dataset nrGIS-40 was 0.72, clearly higher than that of closeness (0.61).

### Performance of Predictions at Various Probability Score Levels

**Figure 5:**
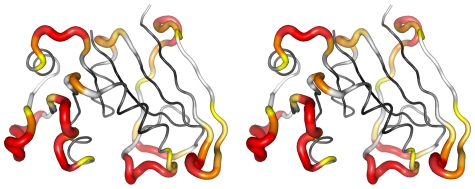
Probability Scores of DHFR. The structure of the dihydrofolate reductase from *Escherichia coli* (PDB entry: 1RX4) is shown as a cross-eye stereo image, in which the thickness of backbone of a residue is in proportion to the probability score computed by our prediction system for that residue. In addition, probability scores are color-coded — a color closer to red represents a higher score. Gray- to black-colored residues have scores increasingly lower than 0.5. Among the 67 residues with probability scores ≥0.5, only 6 are inviable CP sites (shown in blue). The other 61 residues are experimentally-verified viable CP sites [29]. Thus, at a probability score threshold of 0.5, the precision of the developed prediction system for this independent evaluation dataset is 90% (61/67).

In order to draw a clear map for bioengineers and experimenters to apply the developed system, information retrieval experiments were performed to examine the precisions of predictions at various decision thresholds of the probability score. Table 3 and Table 4 demonstrate that, a high threshold of probability score would retrieve fewer residues but obtain a higher proportion of correct predictions than a low threshold would. No matter in Dataset T or the independent DHFR dataset, any residue with a probability score ≥0.75 was an actual CP site (*i.e.*, precision = 1); besides, over 90% of the residues possessing probability scores ≥0.6 were viable CP sites (precision ≥0.9). Since ∼80% of the residues predicted as viable CP sites (*i.e.*, probability scores ≥0.5) in these two datasets were actual CP sites, this system is quite reliable. For those experimenters who expect a high certainty about the viability of the permutants, residues with probability scores ≥0.75 can be good choices for performing CP; at this threshold, only 11%–15% of all residues in a protein will be predicted as viable CP sites. See Figure 5 for a stereo display of the prediction results of DHFR.

**Table 3:** Performance of Predictions for Dataset T at Various Decision Thresholds of the Probability Score.

Probability score	PPF	Recall	Precision	Num of viable CP sites among the retrieved residues	Num of retrieved residues
≥0.80	0.09	0.21	1.00	16	16
≥0.75	0.15	0.34	1.00	26	26
≥0.70	0.21	0.45	0.92	34	37
≥0.60	0.35	0.71	0.89	54	61
≥0.50	0.50	0.91	0.78	69	88
≥0.40	0.57	0.97	0.73	74	101
≥0.30	0.65	1.00	0.66	76	115
≥0.20	0.76	1.00	0.57	76	133
≥0.10	0.86	1.00	0.50	76	152
≥0.00	1.00	1.00	0.43	76	176

**Table 4:** Performance of Predictions for the DHFR dataset at Various Decision Thresholds of the Probability Score.

Probability score	PPF	Recall	Precision	Num of viable CP sites among the retrieved residues	Num of retrieved residues
≥0.80	0.06	0.10	1.00	9	9
≥0.75	0.11	0.20	1.00	17	17
≥0.70	0.19	0.35	0.97	30	31
≥0.60	0.32	0.56	0.94	48	51
≥0.50	0.42	0.71	0.91	61	67
≥0.40	0.54	0.83	0.83	71	86
≥0.30	0.62	0.90	0.78	77	99
≥0.20	0.74	0.97	0.70	83	118
≥0.10	0.96	1.00	0.56	86	153
≥0.00	1.00	1.00	0.54	86	159

### Large-scale Predictions of CP Viability for Known Protein Structures

The amount of protein structural data is rapidly increasing. As of the date of this article, there have been over 70 thousand protein structures, which consist of 177 thousand polypeptide chains, deposited in the PDB [72]. However, the number of unique structures remains relatively stable. Currently the 40% sequence identity non-redundant subset of PDB contains approximately 9,000 polypeptides, as reported by the PDB-REPRDB web server [73]. To stimulate the application of CP in research and biotechnology, this work has performed large-scale predictions for all these 9 thousand representative PDB structures. The results are summarized in Text S1. For every residue in these proteins, the probability score for its CP viability is provided along with its amino acid and secondary structure information. If a residue is a known CP site recorded in CPDB [33] or any of the datasets utilized in this report, it is clearly annotated. Several interesting observations have been made from these predicted and previously identified data.

First, under appropriate conditions, CP can be introduced into a protein at a position within a helix or a sheet-forming strand, or even at a disulfide-bridged Cys. Although our statistics revealed that the occurrence frequencies of CP in such locations are significantly lower than those in the background, these locations are not completely disallowed for CP. There were respectively 16% and 27% residues with α-helix or β-strand conformations in the representative PDB structures predicted to be viable CP sites; in addition, 22% of the disulfide-bridged Cys residues in those protein structures were predicted permissible for CP. To our knowledge, currently among all engineered CPMs, the CPM 293 and CPM 311 of lipase B from *Candida antarctica* (CALB) are the only cases that were created at disulfide bond-forming Cys. These Cys CPMs retained lipase activities [12]. The probability scores calculated by the developed prediction system for these Cys residues were 0.78 and 0.77, respectively (PDB entry: 1TCA; sharing ≤7% sequence identities with the training Dataset T). In addition to these two Cys, the CALB possesses other 61 experimentally-verified viable CP sites [12]. The developed system correctly predicted 84% of these viable CP sites, 18 out of which have helix or strand conformations (see Text S1).

Second, our viable CP site prediction system may also be feasible to suggest functional CP sites. Out of the 13 engineered and functional CPs summarized in the Table 1 of [10], 10 were predicted as viable CPs; for the rest three CP sites, two actually had probability scores close to 0.5 (>0.46; Test S1). Most of these functional CPs were created with a polypeptide linker to connect their native termini. After *in silico* adding the corresponding linkers to the native proteins of these permutants and performing MD simulations, 12 CP sites had probability scores >0.52. In the case of CALB, since all the 63 experimentally-verified permutants were functional [12], the high sensitivity (0.84) of our CP viability prediction system also exemplifies the feasibility of this system for predicting functional CPs.

Third, a viable CP site may also be a good protein splitting or domain insertion site. Protein fragment complementation assays (PCA) involve splitting a certain reporter protein (*e.g.*, an enzyme or a fluorescent protein) into two fragments that cannot function along but are capable of re-assembling and restoring native functions upon being brought close enough. PCA has been widely applied in many protein-protein interaction studies. Among the commonly-used reporter proteins, inteins are also capable of protein trans-splicing and are thus utilized in many protein engineering works [74], [75], [76]. Domain insertion splits a host protein into two parts, between which a foreign domain/protein is inserted [27], [28]. Well-defined protein splitting sites for PCA (see [77] for a summary), protein trans-splicing [75], [76] and domain insertion [28] include residue 35 of ubiquitin (PDB entry: 1UBQ), 197 and 198 of β-lactamase (PDB entry: 1TEM), 106 of DHFR, 102 and 123 and 131 of intein (PDB entry: 2KEQ), 438 of firefly luciferase (PDB entry: 1BA3), 158 of green fluorescent protein (GFP; PDB entry: 1GFL), and 145 of enhanced yellow fluorescent protein (PDB entry: 1YFP). Except Asn 131 of intein (probability score = 0.47), all these residues were predicted by our system as viable CP sites (Test S1) with high probability scores (mean = 0.70). Since viable CP sites are not critical to protein folding [28], these results are reasonable and are helpful to expand the application of the developed CP viability prediction system.

### Relationships between CP sites and Active Sites

CP has been applied to determine the residues important to protein folding or stability [17], [19], [23], [25], [29]. It may thus be supposed that CP is also applicable to probe the active sites of a protein. If this idea is true (*i.e.*, creating CP at an active site residue would inactivate the protein), a method developed for detecting active sites could be reversely used to detect viable CP site. Perhaps this is the reason that the active site predicting measure closeness [59] was eventually used to predict viable CP sites [30]. However, the equivalence between residues important to protein function and residues important to folding has not been established yet from the point of view of structural biology. Comparing the catalytic and ligand binding residues with the CP site residues of DHFR (see Table S1), we found that only 21 out of the 33 active site residues were inviable CP sites, that is, important residues to protein folding. This result demonstrates that determining active sites and determining viable/inviable CP sites can be very different topics, and it may explain why applying closeness to the prediction of viable CP site only achieved an AUC of 0.7 [30]. Therefore, the relationships between CP sites and active sites may not be interpretable directly based on the results of folding researches and are definitely worth further study.

### Importance and Applications

#### Novel Bifunctional Proteins

An interesting application of CP is to create fusion proteins in which the junction of proteins differs from the native termini [26], [27], [28]. When designing fusion proteins, the positions suitable for creating new termini to connect two proteins can be quite limited owing to steric hindrance. Moreover, in previous work CP has been almost exclusively targeted in loops. This bias might considerably reduce the chances of creating products with desired properties. The sequence and structural preferences of CP reported here along with the convenient probability score generated by our prediction system can broaden the choices of CP sites and potentiate the production of many novel fusion CPMs.

#### Protein Folding Studies

CP has long been applied to study protein folding. In reality, the purpose of Iwakura's systematic CP experiments on DHFR was to determine its folding elements, in which any introduced CP is inviable [29]. The ability of our system to predict CP sites implies that it can be used in reverse to predict folding elements. For example, 67 of the 73 residues (*i.e.*, 92%) in folding elements of DHFR were predicted to be inviable CP sites. Iwakura et al. indicated that there are three residues involved in early folding events of DHFR but not located in the folding elements (Val 75, Leu 156, Glu 157 [29]; see also [78]). These residues were viable CP sites but might be still be important to the native folding process of DHFR. Interestingly, these residues were predicted as inviable CP sites by our system (probability scores ≤0.41). For the 10 early folding sites of DHFR that were located within folding elements [29], [78], their probability scores as viable CP sites were even lower (mean = 0.19; maximum = 0.36). Hence, we suggest that the prediction of CP viability by our system may also be relevant to studies of protein folding.

#### Protein Engineering

Improving protein function is an important application of CP. Because being viable are prerequisites for protein function, our viable CP site prediction system, in conjunction with established structural/biochemical information for individual proteins, will help determine candidate CP sites that may result in functionally improved CPMs. For instance, in the case of lipases, which exhibit poor activity and enantioselectivity toward bulky substrates, Qian and Lutz utilized CP to improve the ability of CALB to act on bulky benzoate esters based on the idea that relocation of the protein termini in or near the active site pocket can increase local chain flexibility and thus active site accessibility [12]. Indeed, CPMs created at Ser 150, Ala 283, Ala 284, and Pro 289 were found to have *k*_cat_/*K*_M_ values higher than the wild-type enzyme for certain bulky substrates; these positions are all close to the active site pocket [12]. These activity-improving CP sites are predicted viable by the developed system (Test S1). Thus, our method holds promise for protein engineering by identifying potentially functional CP sites as well as screening out less-probable candidates.

### Conclusions and Future Work

After examining numerous propensities and properties of known CP sites, CP was found to prefer: (1) Gly, Pro, Asp, Asn, and other hydrophilic/neutral residues, especially those with negatively charged or polar uncharged side chains, (2) residues in coils, loops, and turns, and (3) residues with large solvent-accessible surface areas, short distances to the protein surface, few hydrogen bonds, unpacked environments, and high flexibility. By contrast, CP disfavors: (1) Cys, Met, Leu, Ile and other hydrophobic residues, particularly those with bulky or aromatic side chains, (2) residues in helices and β-bridges, and (3) residues in or close to a protein's buried core. An effective CP viability prediction system has been developed based on these identified preferences and four machine learning methods. Using this system, the predicted CP viability of a residue is highly dependable as long as the probability score of the residue is >0.75. Large-scale CP site predictions for representative protein structures were performed, and several additional applications of the developed CP viability prediction system were thus identified. The statistical data, the developed prediction system, and the large-scale prediction results provided in this study will facilitate the application of CP to fundamental research and biotechnology.

The probability score designed in this work was to judge the feasibility for creating viable CPMs. In the future, functional assay data about known CPs will be collected to develop a new score that will describe the probability of creating a functionally improved CPM. As for the current system, because we utilized many published programs that were composed using various computer languages, it is impractical to combine and release them as a single standalone package. Instead, a web server would be an easier way for users to access it. To cope with the heavy computational loads caused by several structural measures and the time-consuming data flow through numerous prediction models, a parallel computing environment is now being constructed for implementing our system into a quick-response web server.

## Materials and Methods

Experiments were performed using 10 Linux computers each with two 2.27-GHz Intel processors and 32 GB of RAM. The sources of protein structure files were snapshots of the PDB and the SCOP from August 2010. In-house programs were written in the C++, PHP, Perl, Python, and R languages. All the published algorithms or publicly available software were applied with default parameters and settings unless otherwise specified.

### Preparation of Experimental Datasets

#### Dataset L, a Literature-derived Dataset

Seven proteins with both experimentally verified viable and inviable CP sites were retrieved from the CP-related literature database provided by CPDB [33]. They were DHFR (PDB entry: 1RX4), disulfide oxidoreductase DsbA (PDB entry: 1A2J), wild-type GFP (PDB entry: 1GFL), GFP superfolder (PDB entry: 2B3P), GFP folding reporter (PDB entry: 2B3Q), myoglobin (PDB entry: 5MBN) and phosphoribosylanthranilate isomerase domain (ePRAI; PDB entry: 1PII, residue: 256–452). Circular permutants of these proteins were generated by using genetic techniques involving duplicated protein genes, circularized DNAs, etc (see references provided below). For DHFR, myoglobin, and ePRAI, permutation sites leading to foldable variants were considered as viable CP sites [15], [29], [79]. Regarding DsbA, viable CPMs referred to those functional CP variants without extensions or deletions [80]. As for the GFPs, CPMs with soluble fraction >5% and relative fluorescence strength >5% of the control set were considered to be viable [81], [82], [83], because the authors of [83] noted that the uncertainty of their experimental data was ∼5%. The CP sites of these proteins that resulted in non-foldable, barely soluble, non-functional, or non-expressible variants were treated as inviable CP sites. These proteins and their 335 viable/inviable CP sites constituted Dataset L (see Supporting Information files). This dataset contained similar amounts of positive and negative data, *i.e.*, 162 viable and 173 inviable CP sites.

#### Dataset T and the DHFR Dataset

For unbiased evaluations of the developed prediction system, before carrying out machine learning procedures Dataset L was divided into two subsets, *i.e.*, Dataset T and the DHFR dataset. This DHFR dataset (86 viable and 73 inviable CP sites) was actually the same as the DHFR dataset established by Iwakura et al. [29], [30]. Dataset T (76 viable and 100 inviable CP sites) was composed of the other 6 proteins from Dataset L. Dataset T and the DHFR dataset shared very low (<9%) sequence identities; the former was used to train and test our prediction methods and the latter was utilized as an independent evaluation dataset.

#### nrCPDB-40 and nrGIS-40: Non-redundant Subsets of the CPDB and GIS

CPDB [33] is a database of proteins with machine-retrieved and manually-verified circular permutants, the majority of which are naturally occurring CP cases. All protein sequences recorded in CPDB were reduced to a 40% sequence identity non-redundant subset by using CD-HIT 4.0 [84]. Afterward, sequences sharing >40% identity with any protein in Dataset L were removed from the reduced dataset by cdhit-2d [84], a dataset comparing program. The remaining 1,059 sequences then formed nrCPDB-40 (see Dataset S2 for a full list). Since all CP pairs remaining in the nrCPDB-40 have low sequence identities, they are thus improbable to be engineered or post-translationally modified CPs but supposed to be the results of complicated evolutionary processes, *e.g.*, duplication/deletion [3], [4] or fusion/fission [5], [6] events. Similar procedures were performed on GIS [35], generating a 40% identity non-redundant subset that also shared <40% sequence identities with Dataset L. This non-redundant GIS subset was then processed with cdhit-2d to filter out any sequences sharing >40% identity with nrCPDB-40. The remaining 2,814 sequences were collected into nrGIS-40 (listed in Dataset S3). Finally, any two datasets among nrCPDB-40, nrGIS-40, Dataset T and the DHFR dataset shared <40% sequence identities.

Note that GIS is a database of structurally similar domains with either co-linear or non-sequential SSE equivalences rather than a specific database of CP-related homologous proteins. Therefore, before utilizing GIS, protein homologs without CP relationships should be eliminated. We downloaded the whole GIS (release v3.04; 24.7 million pairs of structurally similar domains) and screened out homologs without CP relationships according to the GIS annotations. The remaining 3.8 million pairs of CP-related homologous domains were then subjected to structural alignment by GANGSTA+, *i.e.*, the alignment engine of GIS [34], [35], to calculate their structural similarities, inclusive of the alignment size and the RMSD (root-mean-square distance) of structural superimposition.

GANGSTA+ was not capable of distinguishing global CP (the unit undergoing CP is the whole protein) and partial CP (the CP is within a region of the protein); however, the distinction between them can be very critical to CP researches. It has been argued that a partial CP should be considered as a structural “swap” rather than a “circular permutation” [5]. Topologically, a partial CP (*e.g.*, *A*_1_ *A*_2_ *A*_3_ *A*_4_ *A*_5_ *A*_6_ *A*_7_ versus *A*_1_ *A*_2_ *A*_5_ *A*_3_ *A*_4_ *A*_6_ *A*_7_, involving at least three “cut-and-paste” steps) is a much more complicated rearrangement than a global CP (*e.g.*, *A*_1_ *A*_2_ *A*_3_ *A*_4_ *A*_5_ *A*_6_ *A*_7_ versus *A*_5_ *A*_6_ *A*_7_ *A*_1_ *A*_2_ *A*_3_ *A*_4_, involving just one “cut-and-paste” step). Therefore, partial CP would be more appropriately considered a type of “scrambled permutation” [85]. Because here we focused on the type of viable CP that can be artificially created by just a few DNA-leveled “cut-and-paste” engineering steps (see [12], [23], [29], [80] for such genetic engineering protocols), most partial CPs were also filtered out in this study. First, following the settings of several previous CP-related studies [10], [32], CP pairs with size difference ≥50% were excluded from the remaining 3.8 million pairs. Second, homologous pairs with GANGSTA+ alignment size <75% were excluded. Although these two steps did not guarantee the complete clearance of partial CPs, they efficiently removed most of them and reduced the number of homologous pairs to 1.6 millions, which involved 144.9 thousand domains. After further eliminating domains with chain breaks or missing residues and running CD-HIT and cdhit-2d to remove redundant sequences as stated above, the nrGIS-40 was established.

#### nrCPsitecpdb-40 and nrCPsitegis-40: Non-redundant Datasets of CP Sites

In CPDB, every CP site of a protein pair was preliminarily identified by CPSARST [10] and then refined by the theoretically most accurate CP site identification algorithm, *i.e.*, shifting the permutation site residue by residue around the preliminary site to determine the best alignment for the two proteins [6], [32]. The structural alignment engine FAST [86] was used to refine the location of CP sites in CPDB [33]. Differently, GIS neither provided information nor performed any refinement on the CP sites. Hence, in this study, each CP site in GIS was parsed from the alignment output of GANGSTA+ and then refined by the same theoretically most accurate algorithm. To prevent introducing possible bias by the alignment engine, instead of FAST we used TM-align [87] as the alignment engine when refining the locations of CP sites in GIS.

Next, each CP site of nrCPDB-40 was represented by a 20-residue segment that included 10 upstream (toward the N-terminus) and 10 downstream residues relative to the cleavage point. The nrCPsite_cpdb_-40 was then established by applying CD-HIT to reduce the representative segments into a 40% sequence identity non-redundant subset. The same procedure was used to extract the nrCPsite_gis_-40 dataset from nrGIS-40. Detailed lists of nrCPsite_cpdb_-40 (1,087 CP sites) and nrCPsite_gis_-40 (2,718 CP sites) sequences can be found in Datasets S4 and S5.

### Bootstrap Aggregating Analyses of the Occurrence Frequencies of Various Amino Acids in CP Site Representative Segments

To select a suitable length of polypeptide segment to represent a CP site, bootstrap aggregating experiments were performed to preliminarily analyze the amino acid compositional preferences of the CP site representative segments of various lengths. Bootstrapping, a general approach to statistical inference, is a modern random sampling method that helps to estimate the properties, *e.g.*, the standard deviation and the distribution, of a statistic by allowing one to calculate many alternative versions of the statistic that would ordinarily be computed from only one sample [37]. The purpose of utilizing bootstrapping in this experiment was to observe the standard deviation of the average occurrence of each amino acid in the background protein sequences (comparison group) and the CP site representative segments (experimental group). The core algorithm of bootstrap aggregating, given an original dataset *D* of size *n*, is to generate *m* subsets *D_i_* of size *n′*≤*n* by sampling examples from *D* uniformly and with replacement. In our implementation, *m* was set to 5,000. Detailed steps are listed below,

1. Let *D* be the comparison group, *e.g.*, nrCPDB-40, which contains *n* proteins.
2. For each protein *x* in *D*, compute the proportion of each of 20 amino acids.
3. Generate *m* bootstrap samples, each possessing *n′* proteins randomly selected from *D* with replacement, where *n′* = *n*.
4. For each bootstrap sample *D_i_*, the average occurrence of each amino acid is given by,

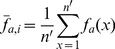
(5)where *a* represents one of the 20 amino acids, *f_a_*(*x*) is the occurrence of *a* in protein *x*, and 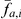 denotes the average occurrence of *a* in *D_i_*.

1. Calculate the standard deviation of the statistic 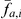 for each amino acid.
2. Then, let *D* be the experimental group, *e.g.*, nrCPsite_cpdb_-40, which contains *n* CP site representative segments each having 2*k* residues. Repeat steps 1–5.

By decreasingly setting *k* from 10 to 1, the average occurrence of the 20 amino acids in CP sites was monitored. As shown in Figure S2, the standard deviations of the frequencies of average amino acid occurrence of the background were all quite small. The difference in amino acid occurrences between the background and CP sites became more obvious as *k* decreased, although the standard deviation of the average amino acid frequencies of the CP sites increased as well. Considering simultaneously the results of these bootstrap aggregating experiments and the coverage analyses of various sequence or secondary structural patterns (Table S1), the segment length of 6 residues (*k* = 3) was determined to represent a CP site in this study.

### Propensity Analyses of CPs by Permutation Tests

A permutation test is a statistical significance test based on random resampling. It has many advantages over a *t* test. For example, it works with the statistic directly and does not require standardization of the statistic [37]. Particularly, even without a normal distribution of the statistic, permutation tests give accurate *p-*values [37]. Here we used permutation tests to examine the significance of differences in primary and secondary structural compositions between the background (whole proteins) and CP sites. Taking the example of the di-residue SSE pattern HH, *i.e.*, two consecutive residues with helical conformation, the frequency of HH in each polypeptide *x* (either a protein or a CP site representative segment) was calculated and denoted as *f*_HH_(*x*). The null hypothesis *H_0_* was that the propensities of HH in the background and CP site groups were not different. To apply resampling, the difference between the sample means was used as a measure of the difference in propensity of HH, that is,
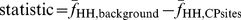
(6.1)

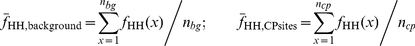
(6.2)where *n_bg_* and *n_cp_* were the respective amounts of polypeptides in the background and CP site groups. The procedure of the permutation test is outlined as follows.

1. Let *N* = *n_bg_*+*n_cp_*.
2. Choose *n_cp_* of the *N* polypeptides at random without replacement to be the new CP site group; the other *n_bg_* polypeptides form the new background group. Calculate the mean frequency of HH in each group and the difference between these means — that is, our statistic.
3. Repeat the above resampling step *T* times to obtain a permutation distribution of the statistic, which estimates the sampling distribution when *H_0_* is true.
4. Let 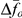 represent the value of the statistic actually observed in the original background and CP site groups. Locate the 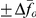 values on the permutation distribution, and determine the number of resampling rounds that yield values equal to or between 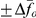. Let *t* denote this number.
5. The permutation test estimate of the *p-*value is given by,

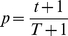
(7)


In this study, the resampling step was repeated *T* = 99,999 times in each permutation test.

### Calculation of Structure-derived Measures

The teLeap program of the AmberTools package [88] was used to add/restore hydrogen atoms to the PDB structure files as necessary. For some special cases to which teLeap failed to add hydrogen atoms, the reduce program [89] was utilized instead. RSA, residue depth, number of hydrogen bonds, and GNM-F were calculated by naccess [90], DPX [56], LIGPLOT [91], and pygnm [92], respectively. GROMACS [93] was used for MD simulations and to obtain the RMSF values. Certain measures were computed following previous studies: closeness [30], [59], CM [57], CN [61], and WCN [64]. The parameter settings for GROMACS in this work is provided in Text S2.

### Assessment of the Dataset Independence of Features

#### Primary and Secondary Structural Features

The primary and secondary structural propensity scores were previously obtained from nrCPDB-40 and assessed here by performing 10-fold cross-validated ROC curve analyses on Dataset T. In each ROC analysis, the stringent binary classification quality measure Matthews correlation coefficient (MCC), which ranges from +1/−1 (perfect/inverse prediction) to 0 (random prediction), was calculated. As listed in Table S3, the binary classification performances of the secondary structural propensity scores (average MCC = 0.49) were generally higher than those of the primary structural propensity scores, whose average MCC (0.27) was still clearly better than that of a random prediction.

#### Tertiary Structural Features

For each tertiary structural feature, the decision threshold between viable and inviable CP populations was determined by ROC analysis based on Dataset T; large-scale predictions were then made for all proteins in nrCPDB-40 to compute the sensitivity, *i.e.*, the true positive rate. Although a high sensitivity meant many known viable CP sites were correctly predicted, it did not necessarily mean a good prediction performance if the true negative rate could not be computed. Unfortunately, true negative rate was inaccessible here because nrCPDB-40 did not possess any negative data. Therefore, the predicted positive fraction (PPF) was calculated. The PPF was defined in this work as the number of residues predicted as viable CP sites divided by the total number of residues contained in the testing dataset. For instance, nrCPDB-40 totally had 212,710 residues; when there were 21,271 residues of nrCPDB-40 predicted to be viable CP sites, the PPF would be 0.1, or 10% (21,271/212,710). For every feature, we adjusted the decision threshold such that the PPF was fixed to 0.5. This PPF level, at which 50% of all residues were predicted as viable CP sites, served as a fair foundation for accessing various features under this no-negative-data scenario. This fixed value of PPF (*i.e.*, 0.5 or 50%), was chosen because a random prediction at 0.5 PPF would theoretically result in a sensitivity of 0.5 in our experiments, as shown by the results of the Rand feature in Table S2. Therefore, if a feature achieved a sensitivity value higher than 0.5 at this PPF, the prediction performance of the feature is better than a random prediction. In this report, wherever a large-scale prediction sensitivity for nrCPDB-40 or nrGIS-40 is mentioned, it means the sensitivity obtained at 0.5 PPF.

As listed in Table S3, the sensitivities for all selected tertiary structural features were ≥0.53. Now that all selected features were trained and tested with very different datasets and the binary classification qualities were acceptable, they were feasible to predict CP sites without a substantial dependence of the performance on the dataset.

### Hierarchical Integration Procedure

To combine the binary classification power of various features, we developed an HI procedure:

1. Hierarchically classify features into a rooted tree according to their characteristics and biological meanings.
2. Starting from the most distant nodes, *i.e.*, branch points, from the root, features sharing a common node are integrated using the following formula,

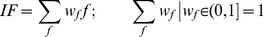
(8)where *IF* stands for the integrated feature, *f* denotes a component feature of *IF*, and *w_f_* is the weight given to *f*. Before the integration, each component feature should be standardized using Formula 4.

1. For each node, generate all possible combinations of *w_f_* values for *IF*. In this work, each *w_f_* value had two decimal places.
2. Determine the optimal weights for all component features of *IF* based on the binary classification quality and statistical significance of *IF*. In this work, the order of the considered quality measures was the 10-fold averaged MCC, 10-fold averaged AUC, and *p-*value obtained with Dataset T.
3. Repeat steps 2–4 until the root *IF* is optimized.

In this procedure, the first step should be performed manually based on sufficient background knowledge about the features. Steps 3 and 4 are actually an exhaustive search for optimal weights. Because each node in our feature classification tree (see Figure 4) had only a small number of branches, it was not very time-consuming to perform this exhaustive search.

### Application of Conventional Machine Learning Methods

#### ANN

We utilized a three-layered ANN (input, hidden, and output layer) with a sigmoid activation function and the back-propagation learning algorithm [94], [95]. The C++ code of ANN written by Paras Chopra and the Python code of the back-propagation algorithm written by Neil Schemenauer were integrated and rewritten into an object-oriented PHP program. The number of hidden neurons *N_h_* was given by Round(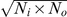), where *N_i_* and *N_o_* are the number of input neurons and the number of output neurons, respectively. The initial weights for dendrites were random values with the range −2 to +2. The learning rate and momentum were respectively set to 0.5 and 0.1. The number of iterations was 5,000. In each iteration, a known case was randomly selected from Dataset T to train the network.

#### SVM

SVM prediction models in this work were established by the LIBSVM package [96] using the regularized support vector classification algorithm (parameter setting: -s 0) and a radial basis kernel function (parameter setting: -t 2). The optimal setting of the penalty cost (parameter -c) and the gamma value (parameter -g) for the kernel function was determined by the program grid.py (with default settings) included in LIBSVM.

#### Decision Tree and RF

An RF consists of many decision trees, each of which is grown using a randomly selected subset of available features and trained with a bootstrap sample of the training dataset. The final output of an RF is determined by a majority vote of individual trees. In this work, each tree was generated using a reprogrammed C4.5 package [97], and the RFs were established using the following algorithm.

1. Let *n_t_* and *n_f_* denote the number of training cases and the number of available features, respectively (*n_t_*>0; *n_f_*>0).
2. Randomly choose *n_f_′* features to grow a tree. If *n_f_*≥2, then *n_f_′*≤0.5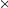 *n_f_*; or *n_f_′* = *n_f_*.
3. Take a bootstrap sample of *n_t_′* cases from the training set, where *n_t_′* = *n_t_*, to train this tree. Theoretically, the expected number of unique cases taken from the training set would be approximately 63%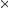 *n*. The rest of the unique cases of the training set (37%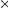 *n* cases) were used to evaluate this tree by predicting their classes, *i.e.*, viable or inviable CP sites, and calculating the MCC.
4. This tree is fully grown and not pruned.
5. Repeat steps 2–4 until 1,000 trees are grown.
6. Sort the trees in descending order according to MCC values.
7. The RF is formed with the 500 trees having the highest MCC values.

### Probability Scores

The purpose of the probability scores designed in this work was not to precisely determine the “probability” of a residue being a viable CP site but rather to provide an easily understandable “score”, which has the range from 0 to 1 and is conceptually in direct proportion to the chance that a residue is permissible for CP.

The output of our ANN predictor was a real number between 0 and 1. It was directly used as a probability score. LIBSVM provides a sophisticated method for calculating the probability estimate [96], which was taken to be the probability score of our SVM model. Because an RF was composed of 500 decision trees, the probability score of an RF model was calculated as the proportion of trees that predicted the residue of interest as a viable CP site. As in the HI models, we used the score distributions of the positive and negative cases of Dataset T as the standard. After obtaining the IF value of the residue of interest (*IF_i_*), the number of residues with IF values ≥*IF_i_* in the standard distribution of positive cases (*N_p_*) and the number of residues with IF values ≤*IF_i_* in the standard distribution of negative cases (*N_n_*) were counted. Then the probability score was calculated as *N_n_*/(*N_p_*+*N_n_*). This procedure is equivalent to considering *IF_i_* as the decision threshold between positive and negative predictions and calculating the proportion of true negatives to true negatives plus true positives, *i.e.*, TN/(TN+TP). Since this proportion ranged from 0 to 1 and increased as the IF value increased, it served as a convenient probability score. Because the output probability score of any predictor in our system is a real number between 0 and 1, the probability scores obtained from various predictors could be simply averaged into a single final score, which also ranges between 0 and 1. To consider the effects of neighboring residues, after computing the raw probability scores a 3-residue weighted window was then applied to smooth the scores. Let *ps* and *ps*′ denote the raw probability score and the smoothed probability score, respectively. The final probability score for residue *i* was given by,
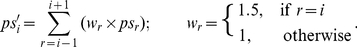
(9)


### Nomenclatural Acts

The electronic version of this document does not represent a published work according to the International Code of Zoological Nomenclature (ICZN), and hence the nomenclatural acts contained in the electronic version are not available under that Code from the electronic edition. Therefore, a separate edition of this document was produced by a method that assures numerous identical and durable copies, and those copies were simultaneously obtainable (from the publication date noted on the first page of this article) for the purpose of providing a public and permanent scientific record, in accordance with Article 8.1 of the Code. The separate print-only edition is available on request from PLoS by sending a request to PLoS ONE, Public Library of Science, 1160 Battery Street, Suite 100, San Francisco, CA 94111, USA along with a check for $10 (to cover printing and postage) payable to “Public Library of Science”.

In addition, this published work and the nomenclatural acts it contains have been registered in ZooBank, the proposed online registration system for the ICZN. The ZooBank LSIDs (Life Science Identifiers) can be resolved and the associated information viewed through any standard web browser by appending the LSID to the prefix “http://zoobank.org/”. The LSID for this publication is: (to be determined by PLoS ONE).

## Supporting Information

Dataset S1 **Dataset L.** This file provides information about how viable and inviable CPs were determined in the Dataset L, which is composed of two subsets: Dataset T and the DHFR dataset.
(XLS)

Dataset S2 **nrCPDB-40.** This file lists the PDB entries for nrCPDB-40.
(XLS)

Dataset S3 **nrGIS-40.** This file lists the SCOP entries for nrGIS-40.
(XLS)

Dataset S4 **nrCPsite_cpdb_-40.** This file provides detailed position and sequence data for nrCPsite_cpdb_-40.
(XLS)

Dataset S5 **nrCPsite_gis_-40.** This file provides detailed position and sequence data for nrCPsite_gis_-40.
(XLS)

Figure S1 **Definition of a CP Site.** This figure is a simple illustration of the working definition of a CP site.
(PDF)

Figure S2 **Amino Acid Compositions of Viable CP Sites and Background Protein Sequences.** (**a**) Absolute occurrence frequency values for 20 amino acids. (**b**) Relative frequency values with respect to the background for 20 amino acids. In this experiment, protein sequences of nrCPDB-40 were utilized as the “background group”. CP site representative sequences of nrCPsite_cpdb_-40 with lengths varied from 20 (±10) to 2 (±1) residues were the “CP site groups”. These results indicate that certain amino acids have increasingly different occurrence frequencies from the background at positions increasingly close to the CP site.
(PDF)

Figure S3 **Distribution of Occurrence Frequencies for 20 Amino Acids.** This figure demonstrates the non-normal distribution of occurrence frequencies of each amino acid. To determine the significance of difference between samples with non-normal distributions, the traditional t-test is inadequate; instead, the permutation test [37] was utilized in this study.
(PDF)

Figure S4 **Sequence and Secondary Structural Propensities of Viable CP Sites in the nrGIS-40 Dataset.** Similar to those in Figure 1, in these charts, each bar shows the relative occurrence of a pattern for the background polypeptides and viable CP sites; but, the background and CP site groups utilized here are nrGIS-40 and nrCPsite_gis_-40, respectively. Patterns examined in this experiment are the same as those shown in Figure 1.
(PDF)

Figure S5 **Propensities of viable CP sites for di-residue, oligo-residue, and residue coupling patterns.** The background and CP site groups of these experiments are nrCPDB-40 and nrCPsite_cpdb_-40, respectively. In each chart, the label for the *x* axis indicates the type of pattern under analysis. Compared with single-residue patterns (Figure 1 and S4), the occurrence frequencies of these di/oligo-residue and residue coupling patterns show larger differences from the background.
(PDF)

Figure S6 **Ramachandran Map and Kappa-alpha Map.** (**a**) The Ramachandran map of SARST [50]. (**b**) The traditional Ramachandran plot. (**c**) The kappa-alpha map of 3D-BLAST [51]. (**d**) The traditional kappa-alpha plot.
(PDF)

Table S1
**Occurrence Coverage of Various Sequence and Secondary Structural Patterns.** A pattern means a specific way of combination of elements. The occurrence coverage of a pattern is defined as the observed number of combinations divided by the theoretical maximum number of combinations. For instance, the di-residue amino acid pattern has 400 (20 elements 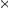
20 elements) possible combinations. If there were only 300 combinations observed in the CP site representative fragments, the occurrence coverage of this pattern was thus 75%.(XLS)

Table S2 **All Features Examined in This Work.** This table summarizes the binary classification performances of all examined sequence, structure and dynamics property measures and the reasons that some of them were excluded from the final feature set.
(XLS)

Table S3 **CP Viability Prediction Performance of the 46 Selected Features.** Each selected feature was subjected to 10-fold cross-validated ROC curve analysis, and was applied to generate a single-feature SVM, ANN and RF predictors. Each predictor was evaluated by 10-fold cross-validation. Dataset T and nrCPDB-40 were utilized in these experiments; in order to assess the dataset independence of each feature, they were applied either to train or test a predictor but not at the same time.
(XLS)

Text S1 **Results of Viable CP Site Predictions for 8,859 Representative Protein Structures.** The developed CP viability prediction system has been applied to predict viable CP sites for a 40% sequence identity non-redundant subset of the PDB. Detailed results are provided in this parser-friendly plain text file.
(TXT)

Text S2 **Parameter settings for the MD simulation package GROMACS.** Parameter settings for the two steps for running simulation with GROMACS, *i.e.*, energy minimization and molecular dynamics simulation, are provided in this plain text file.
(TXT)
