# Supplementary material for: Deciphering the Preference and Predicting the Viability of Circular Permutations in Proteins
Source: PLoS One. 2012 Feb 16;7(2):e31791. doi: 10.1371/journal.pone.0031791 (PMC3281007; doi:10.1371/journal.pone.0031791)
Supplement: Figure S1 — Definition of a CP Site. This figure is a simple illustration of the working definition of a CP site. (PDF) [file pone.0031791.s006.pdf]

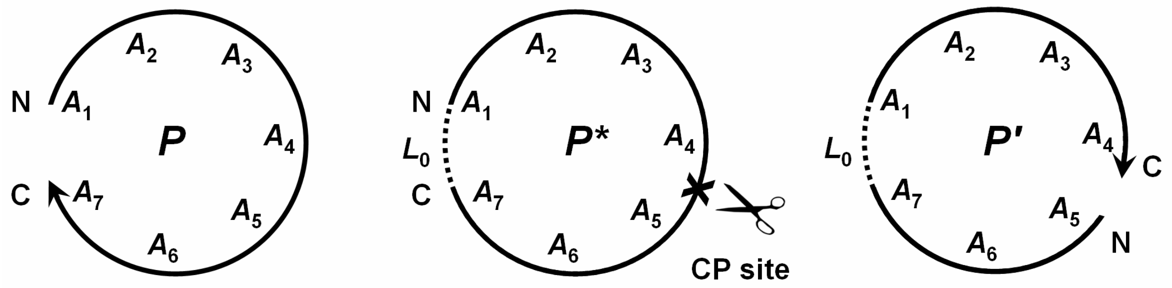

Figure S1. Definition of a CP site. A CP can be visualized as if the native amino (N)- and carboxyl (C)-termini of a protein *P* were linked (*P**) and new ones were created elsewhere (*P′*), resulting a pair of homologs with different locations of termini (*e.g.*, *P* versus *P′*). A polypeptide linker (L_0) may be required to connect the native termini, especially when the termini are distant from each other. A CP site of a protein is defined as a position at which the "cleavage event" occurs; it can be computed as the residue that corresponds to the N-terminal residue of a permutant. In this example, since the residue A_5 of *P* corresponds to the N-terminal residue of *P′*, it is the CP site for generating *P′* from *P*. Note that this figure is a simple illustration of the working definition of a CP site rather than the evolutionary mechanism of CP or an actual artificial procedure for creating a circular permutant. The mechanisms underlying natural CP are not fully understood. Although posttranslational modification may promote CP (see [1] and [Nature (1985) 313: 64-67]), the majority of CPs may result from complex genetic events, such as the proposed duplication/deletion [2,3], fusion/fission [4,5] and other models (see [29] for a summary). See [8,11,25,75,76,77,78,79] for wet-lab protocols for generating artificial circular permutants.
