# Supplementary material for: Deciphering the Preference and Predicting the Viability of Circular Permutations in Proteins
Source: PLoS One. 2012 Feb 16;7(2):e31791. doi: 10.1371/journal.pone.0031791 (PMC3281007; doi:10.1371/journal.pone.0031791)
Supplement: Figure S2 — Amino Acid Compositions of Viable CP Sites and Background Protein Sequences. (a) Absolute occurrence frequency values for 20 amino acids. (b) Relative frequency values with respect to the background for 20 amino acids. In this experiment, protein sequences of nrCPDB-40 were utilized as the “background group”. CP site representative sequences of nrCPsitecpdb-40 with lengths varied from 20 (±10) to 2 (±1) residues were the “CP site groups”. These results indicate that certain amino acids have increasingly different occurrence frequencies from the background at positions increasingly close to the CP site. (PDF) [file pone.0031791.s007.pdf]

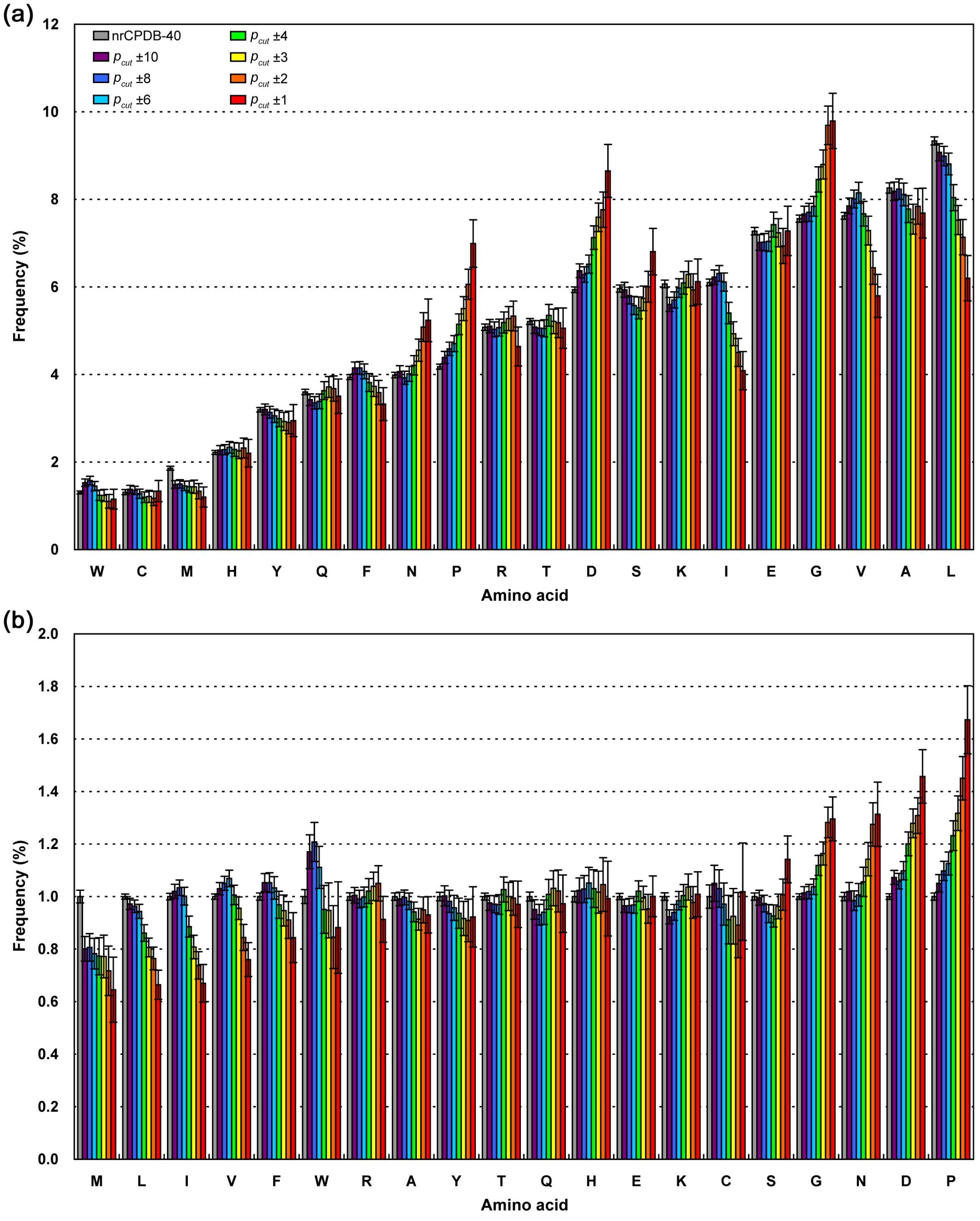

Figure S2. Amino acid compositions of viable CP sites and background protein sequences. In this experiment, protein sequences of nrCPDB-40 were utilized as the "background group" (gray bars). CP site representative sequences of nrCPsite$_{cpdb}$-40 with lengths varied from 20 (±10) to 2 (±1) residues were the "CP site groups". CP site groups are colored with the colors of a rainbow; a warmer color (*i.e.*, closer to red) represents a shorter CP site representative sequence, which covers a smaller section around the CP cleavage point (p_{cut}). **(a)** Absolute occurrence frequency values for 20 amino acids. **(b)** Relative frequency values with respect to the background for 20 amino acids. The error bars represent standard deviations computed by using a random resampling technique known as bootstrap (see **Materials and Methods**). These results indicate that certain amino acids have increasingly different occurrence frequencies from the background at positions increasingly close to the CP site.
