# Supplementary material for: Deciphering the Preference and Predicting the Viability of Circular Permutations in Proteins
Source: PLoS One. 2012 Feb 16;7(2):e31791. doi: 10.1371/journal.pone.0031791 (PMC3281007; doi:10.1371/journal.pone.0031791)
Supplement: Figure S3 — Distribution of Occurrence Frequencies for 20 Amino Acids. This figure demonstrates the non-normal distribution of occurrence frequencies of each amino acid. To determine the significance of difference between samples with non-normal distributions, the traditional t-test is inadequate; instead, the permutation test [37] was utilized in this study. (PDF) [file pone.0031791.s008.pdf]

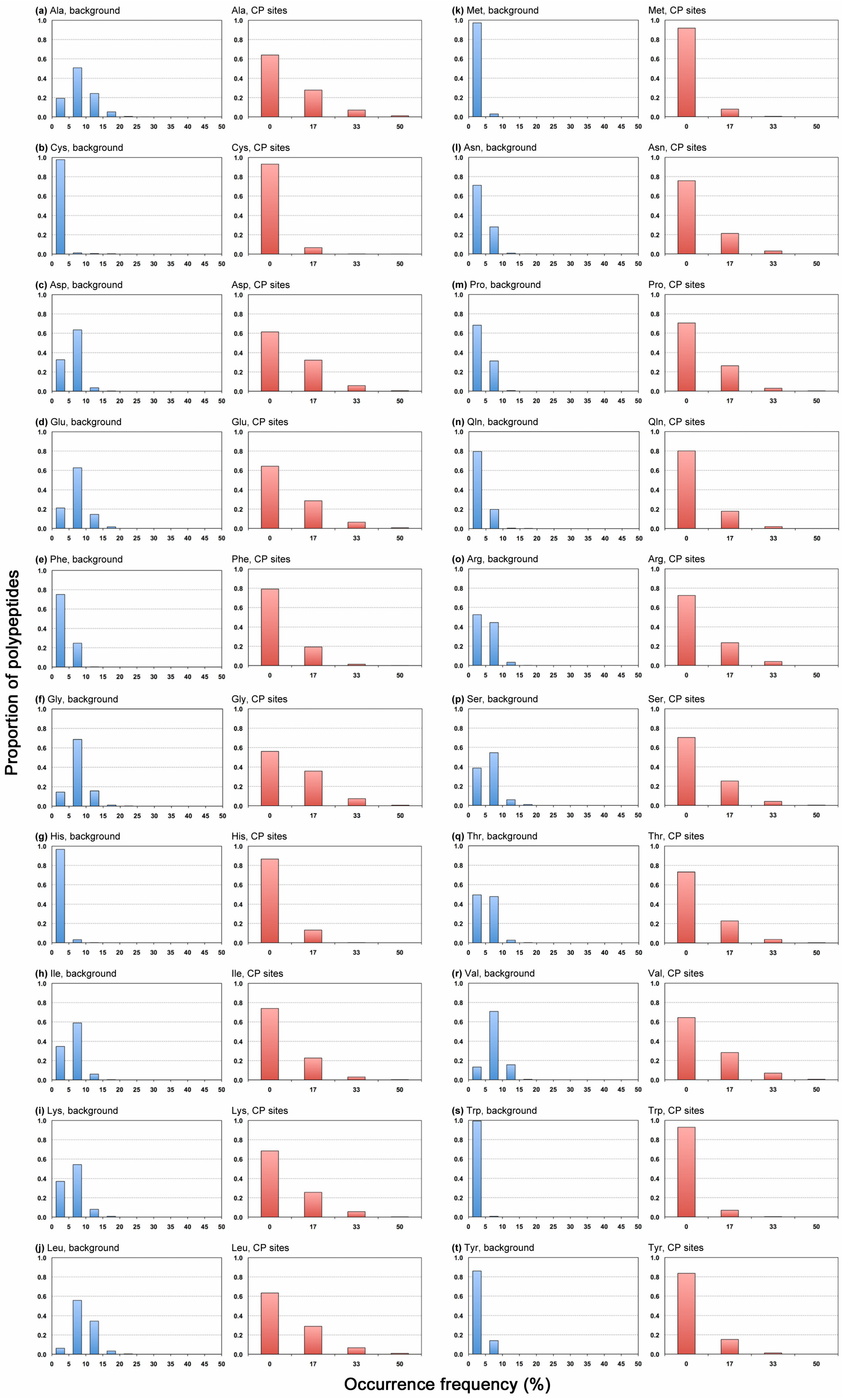

(a) Ala, background
Ala, CP sites
(b) Cys, background
Cys, CP sites
(c) Asp, background
Asp, CP sites
(d) Glu, background
Glu, CP sites
(e) Phe, background
Phe, CP sites
(f) Gly, background
Gly, CP sites
(g) His, background
His, CP sites
(h) Ile, background
Ile, CP sites
(i) Lys, background
Lys, CP sites
(j) Leu, background
Leu, CP sites
(k) Met, background
Met, CP sites
(l) Asn, background
Asn, CP sites
(m) Pro, background
Pro, CP sites
(n) Qln, background
Qln, CP sites
(o) Arg, background
Arg, CP sites
(p) Ser, background
Ser, CP sites
(q) Thr, background
Thr, CP sites
(r) Val, background
Val, CP sites
(s) Trp, background
Trp, CP sites
(t) Tyr, background
Tyr, CP sites
Proportion of polypeptides
Occurrence frequency (%)

Figure S3. Distribution of occurrence frequencies for 20 amino acids. This figure demonstrates the non-normal distribution of occurrence frequencies of each amino acid. The background group (nrCPDB-40; blue bars) and CP site groups (nrCPsite$_{\mathrm{cpdb}}$-40, p_{cut}±3; red bars) were the same as those used in Figure S2. In each diagram, the *x* axis represents the occurrence frequency of a specific amino acid in a polypeptide, and the *y* axis represents the proportion of polypeptides that possessing a certain occurrence frequency of the amino acid. For instance, The first blue bar in diagram (a) indicates that there were 20% (*i.e.*, 0.2) of all polypeptides in the background group that possessed 0% – 5% Ala residues. The distributions of the CP site groups are all skewed to the left and clearly non-normal. Although some amino acids, *e.g.*, Ala and Gly, have a visually more normal distribution than the CP site groups do, most amino acids still have very skewed distributions. To determine the significance of difference between samples with non-normal distribution(s), the traditional t-test is inadequate; instead, we performed the permutation test (see **Materials and Methods**).
