# Supplementary material for: Deciphering the Preference and Predicting the Viability of Circular Permutations in Proteins
Source: PLoS One. 2012 Feb 16;7(2):e31791. doi: 10.1371/journal.pone.0031791 (PMC3281007; doi:10.1371/journal.pone.0031791)
Supplement: Figure S4 — Sequence and Secondary Structural Propensities of Viable CP Sites in the nrGIS-40 Dataset. Similar to those in Figure 1, in these charts, each bar shows the relative occurrence of a pattern for the background polypeptides and viable CP sites; but, the background and CP site groups utilized here are nrGIS-40 and nrCPsitegis-40, respectively. Patterns examined in this experiment are the same as those shown in Figure 1. (PDF) [file pone.0031791.s009.pdf]

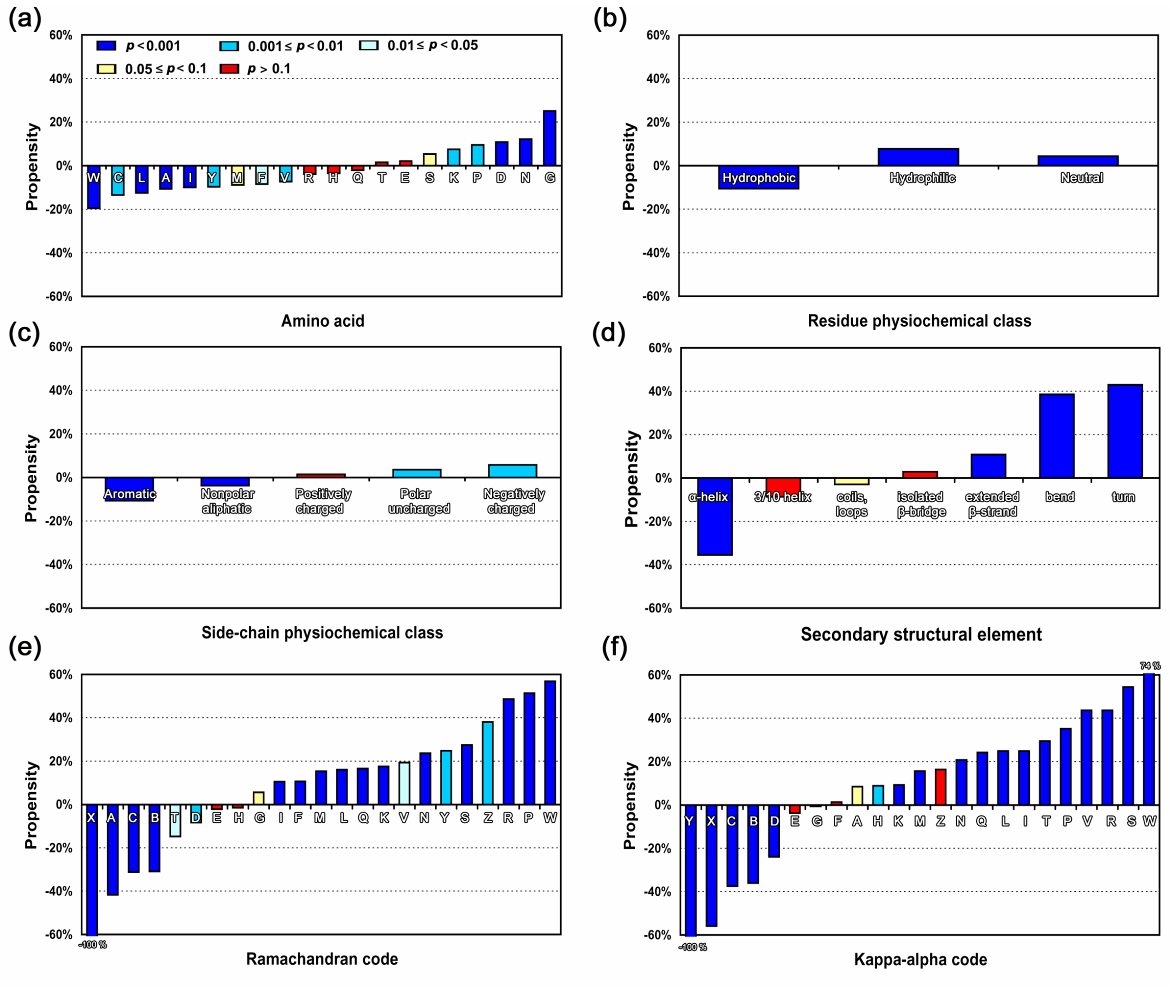

Figure S4. Sequence and secondary structural propensities of viable CP sites in the nrGIS-40 dataset. Similar to those in Figure 1, in these charts, each bar shows the relative occurrence of a pattern for the background polypeptides and viable CP sites; but, the background and CP site groups utilized here are nrGIS-40 and nrCPsite$_{gis}$-40, respectively. The background value was considered as the zero point in each experiment. Dark blue- to light blue-colored bars represent smaller *p*-values (<0.05) for the difference between the background and CP site groups. The yellow- and red-colored bars represent *p*-values ≥0.05. The *p*-values were calculated by permutation test. Patterns examined in this experiment include: **(a)** amino acids, **(b)** residue physiochemical types classified according to [34], **(c)** side-chain physiochemical types classified according to [35], **(d)** SSE determined by DSSP [45], **(e)** Ramachandran code, the backbone conformational alphabet defined by SARST [46], and **(f)** kappa-alpha code, the backbone conformational alphabet defined by 3D-BLAST [47].
