# Supplementary material for: Deciphering the Preference and Predicting the Viability of Circular Permutations in Proteins
Source: PLoS One. 2012 Feb 16;7(2):e31791. doi: 10.1371/journal.pone.0031791 (PMC3281007; doi:10.1371/journal.pone.0031791)
Supplement: Figure S5 — Propensities of viable CP sites for di-residue, oligo-residue, and residue coupling patterns. The background and CP site groups of these experiments are nrCPDB-40 and nrCPsitecpdb-40, respectively. In each chart, the label for the x axis indicates the type of pattern under analysis. Compared with single-residue patterns (Figure 1 and S4), the occurrence frequencies of these di/oligo-residue and residue coupling patterns show larger differences from the background. (PDF) [file pone.0031791.s010.pdf]

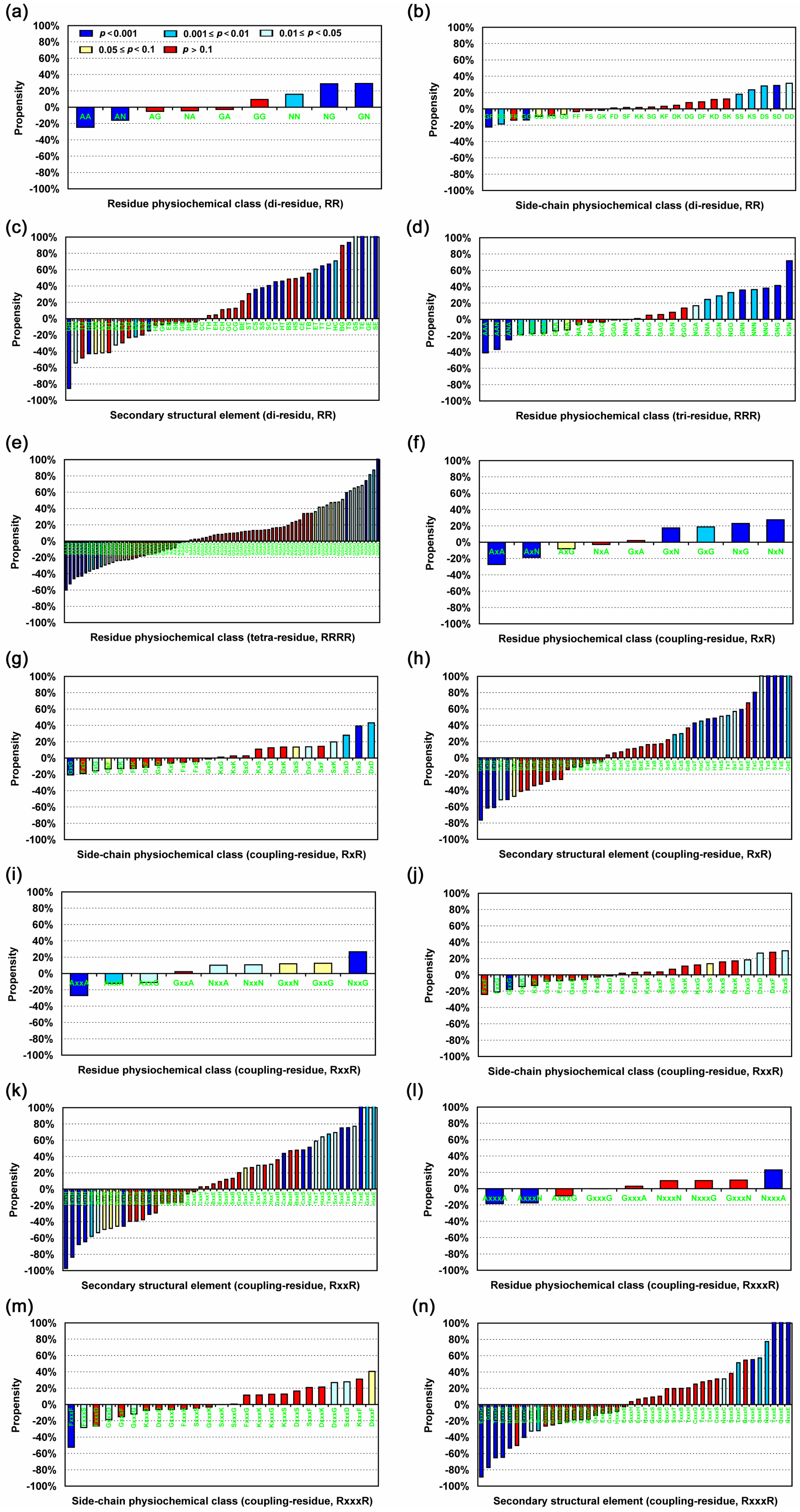
(a)
p < 0.001
0.001 ≤ p < 0.01
0.01 ≤ p < 0.05
0.05 ≤ p < 0.1
p > 0.1
Propensity
AA AN AG NA GA GG NN NG GN
Residue physiochemical class (di-residue, RR)
(b)
Side-chain physiochemical class (di-residue, RR)
(c)
Secondary structural element (di-residu, RR)
(d)
Residue physiochemical class (tri-residue, RRR)
(e)
Residue physiochemical class (tetra-residue, RRRR)
(f)
AxA AxN AxG NxA GxA GxN GxG NxG NxN
Residue physiochemical class (coupling-residue, RxR)
(g)
Side-chain physiochemical class (coupling-residue, RxR)
(h)
Secondary structural element (coupling-residue, RxR)
(i)
AxxA AxxN AxxG GxxA NxxA NxxN GxxN GxxG NxxG
Residue physiochemical class (coupling-residue, RxxR)
(j)
Side-chain physiochemical class (coupling-residue, RxxR)
(k)
Secondary structural element (coupling-residue, RxxR)
(l)
AxxxA AxxxN AxxxG GxxxG GxxxA NxxxN NxxxG GxxxN NxxxA
Residue physiochemical class (coupling-residue, RxxxR)
(m)
Side-chain physiochemical class (coupling-residue, RxxxR)
(n)
Secondary structural element (coupling-residue, RxxxR)

Figure S5. Propensities of viable CP sites for di-residue, oligo-residue, and residue coupling patterns. The background and CP site groups of these experiments are nrCPDB-40 and nrCPsite$_{cpdb}$-40, respectively. The color codes shown in (a) for *p*-values are the same as those of Figure 1. In each chart, the label for the *x* axis indicates the type of pattern under analysis. An R stands for a residue at a position of interest, whereas an x stands for a residue at an uninterested (*i.e.*, to be skipped) position. For example, given an amino acid sequence ARTOFCP, the elements extracted from this sequence that match the pattern RxxR include: AxxO, RxxF, TxxC and OxxP. The fundamental elements used in the experiments for (a), (d), (e), (f), (i) and (l) are the residue physiochemical types classified according to [34]; elements for (b), (g), (j) and (m) are the side-chain physiochemical types classified according to [35]; elements for (c), (h), (k) and (n) are the SSE codes defined by DSSP [45]. Compared with single-residue patterns (Figure 1 and S4), the occurrence frequencies of these di/oligo-residue and residue coupling patterns show larger differences from the background. A lot of information can be extracted from these results. Take (c) for instance. If we consider SSE codes H (α-helix), G (3/10 helix) and I (π-helix) as helices, E (extended strand participating in β-ladder) and B (isolated β-bridge, which forms a single pair β-sheet hydrogen bond) as strands, and T (turn), S (bend) and C (others) as less-regular SSEs, then most of the di-residue SSE patterns (see (c)) that has occurrence frequencies 50% higher than the background represent conformations of the terminal residues of a helix or a strand, such as SE (bend-to-strand), GE (helix-to-strand), TE (turn-to-helix), GS (helix-to-bend) and HC (helix-to-coil). These facts imply that CP tends to occur at the transitional regions between two different regular SSEs (helix/strand), or between a regular SSE and a less-regular conformation.
