# Supplementary material for: Deciphering the Preference and Predicting the Viability of Circular Permutations in Proteins
Source: PLoS One. 2012 Feb 16;7(2):e31791. doi: 10.1371/journal.pone.0031791 (PMC3281007; doi:10.1371/journal.pone.0031791)
Supplement: Figure S6 — Ramachandran Map and Kappa-alpha Map. (a) The Ramachandran map of SARST [50]. (b) The traditional Ramachandran plot. (c) The kappa-alpha map of 3D-BLAST [51]. (d) The traditional kappa-alpha plot. (PDF) [file pone.0031791.s011.pdf]

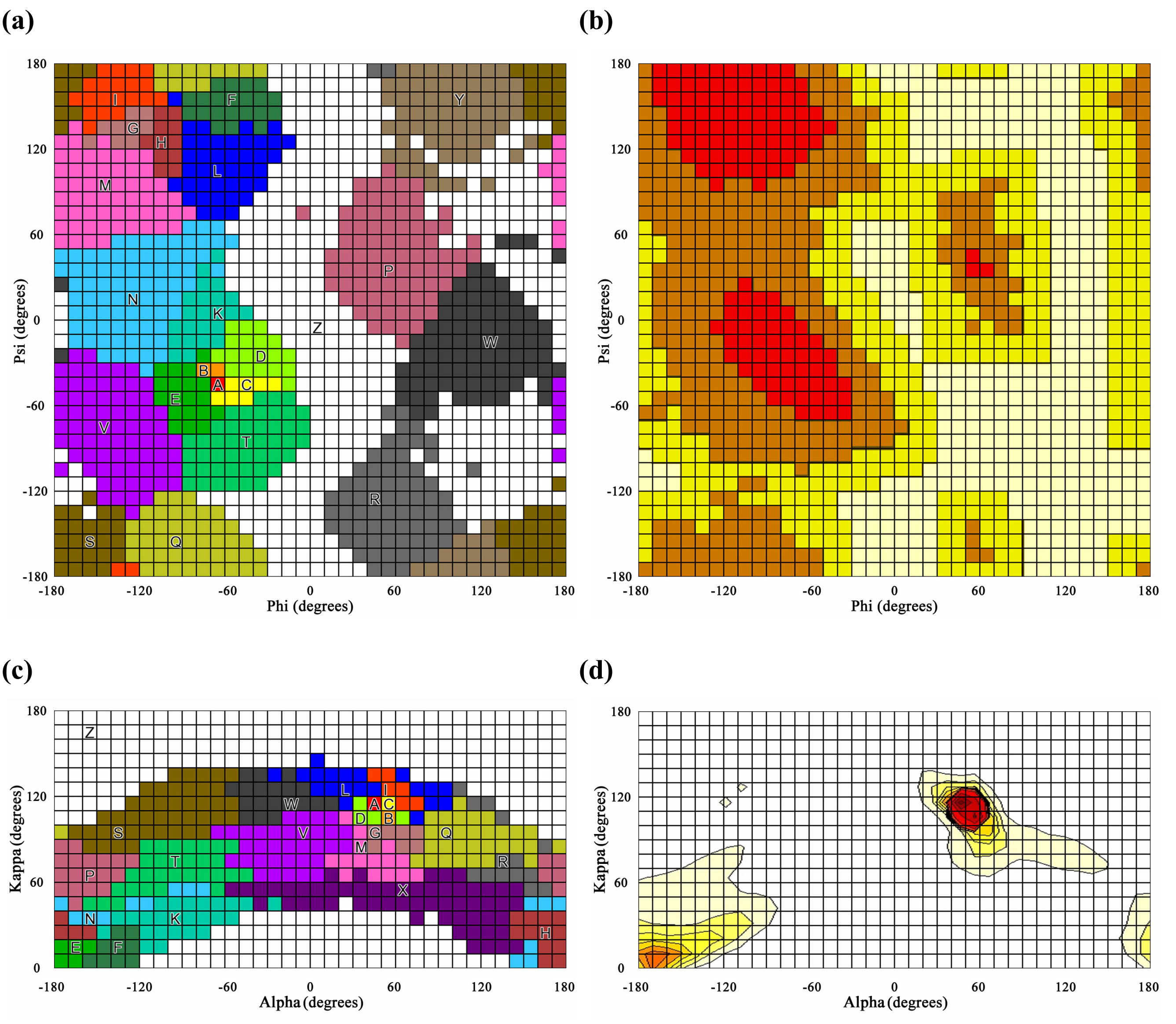

Figure S6. Ramachandran map and kappa-alpha map. (a) The Ramachandran map of SARST [46]. **(b)** The traditional Ramachandran plot [Adv Protein Chem (1968) 23:283-438]. **(c)** The kappa-alpha map of 3D-BLAST [47]. **(d)** The traditional kappa-alpha plot [45]. In (a) and (c), different colors simply represent different code regions. Plots (b) and (d) are in essence contour maps of the density of residues with known conformations; regions with a red background color possess the highest density of residues, implying that the backbone conformations defined by these regions are highly favored. A lighter color in (b) and (d) represents a lower density of residues. Regions with a white background, which are known as the "disallowed regions", consist of very few residues. Different types of secondary structural elements have different preferred regions on these maps. For instance, α-helices mostly occupy the central left red region on the traditional Ramachandran plot, which corresponds to regions A–C and part of regions D, E, T and K on SARST's Ramachandran map (see [46] for preferences of other SSEs). On the traditional kappa-alpha plot, the red regions defined by 96–127° kappa and 36–66° alpha, which largely correspond to regions Y and A–C on 3D-BLAST's kappa-alpha map, exclusively consist of α-helices (refer to [47] for preferences of other SSEs). Certain amino acids also have preferred regions on the Ramachandran plot. See [48] for further information. Note that on the kappa-alpha map of 3D-BLAST, region Y, which is not labeled in this figure because of its small area, is equivalent to the upper right quarter of region A [47].
